# Proceedings from the 2nd Annual UK Implementation Science Research Conference, 'Advancing the science of scaling up: Improving efficiency and effectiveness of implementation strategies in healthcare': meeting abstracts: London, United Kingdom. 18 July 2019

**DOI:** 10.1186/s13012-019-0911-5

**Published:** 2019-07-10

## P1 What makes innovations both ‘stick’ and ‘spread’? A multidisciplinary systematic review to understand implementation depth and scale-up of innovations in healthcare

### Alexandra Ziemann^1^, Yaru Chen^1^, Yiannis Kyratsis^1,2^, Charitini Stavropoulou^1,3^, Harry Scarbrough^1,4^

#### ^1^Centre for Healthcare Innovation Research (CHIR), City, University of London, London, United Kingdom; 2 VU Amsterdam, Amsterdam 1081 HV, Netherlands; ^3^School of Health Sciences, City, University of London, London, United Kingdom; ^4^CASS Business School, City, University of London, London, United Kingdom

**Correspondence:** Alexandra Ziemann (alexandra.ziemann@city.ac.uk)

**Background**

Often, healthcare innovations are not sustained after adoption and vary in their effectiveness when scaled-up.[1] Diverging research strands focus on either implementation or scale-up/diffusion. These strands typically have different analysis levels, focus on different implementation phases and are conducted in different research fields (e.g., health research (implementation), organisation studies (diffusion)). This systematic review aimed at synthesising evidence on implementation depth and innovation diffusion published in these diverging research fields.

**Method**

We systematically searched eleven health and organisation/management studies databases for theoretical and empirical studies published in English language, including Medline and Business Source Complete. The search strategy combined terms for innovation with terms for implementation depth or diffusion. Further we hand-searched key textbooks, references and citations of included studies and relevant journals in the two fields. Two reviewers screened for inclusion and extracted data. Conflicts were resolved in the team. Data are analysed applying qualitative thematic analysis and summarised in a narrative synthesis.

**Results**

Preliminary results show that conceptualisations of implementation depth and innovation diffusion vary according to the theoretical perspective. They can be conceptualised along dimensions of scale (extensiveness, completeness, prevalence), scope (degree of fit, fidelity and adaptation), process (incremental and continuous or disruptive and episodic) and outcome (decoupling and ephemeral or sustained change).

**Conclusion**

The review synthesises concepts from different levels of analysis and diverse literatures. It sheds light on the blind spot in our understanding of how to achieve both, the widespread and sustainable implementation of innovations.

**Acknowledgements**

This abstract is presented on behalf of the interdisciplinary Centre for Healthcare Innovation Research (CHIR) at City, University of London. All authors contributed equally to the abstract.

**Reference**

1. Health Foundation, editor. The spread challenge. London: Health Foundation; 2018.

## P2 Scaling up guidance documents on dementia palliative care: PAR methods and strategies to promote implementation

### Suzanne Timmons^1^, Nicola Cornally^1^, Irene Hartigan^1^, Elaine Lehane^1^, Catherine Buckley^2^, Christina O’Loughlin^4^, Colette Finn^1^, Marie Lynch^3^, Alice Coffey^4^

#### ^1^University College Cork, Cork, Ireland; ^2^St Luke’s Home, Cork, Ireland; ^3^Irish Hospice Foundation, Dublin, Ireland; ^4^University of Limerick, Limerick, Ireland

**Correspondence:** Alice Coffey (alice.coffey@ul.ie)

**Background**

Dementia is a progressive illness and in the later stages, the person will have difficulty communicating their needs in relation to thirst, hunger, pain or discomfort. In Ireland, over one third of people with dementia reside in Long Term Care settings. Evidence based guidance documents for dementia palliative were developed by the authors with the support of the Irish Hospice Foundation. The aim of this study is to implement the guidance intervention in three Long Term Care (LTC) facilities in Ireland.

**Method**

The Consolidated Framework for Implementation Science (CFIR) underpinned the 3 phases to promote implementation. Phase 1 consisted of situational analysis, interviews with residents and carers and audit of current practice to identify factors that for the up scaling of the intervention and implementation process. The implementation process is guided by Participatory Action Research (PAR) methodology. These data informed the design of the educational intervention within work based learning groups (WBLG).

**Results**

Work based learning groups with virtual and physical resources were developed using combined blended approach to learning. Four face-to-face sessions with active and creative learning opportunities were designed based on findings from the situational analysis results in phase 1.

**Conclusion**

This dynamic approach to implementing the guidance within WBLG enables learners to have greater control over how the learning takes place. Provisions within the work place allow for ‘in employment’ education to overcome any disconnect associated with traditional education methods. Using this Participatory Action Research process within an implementation framework created learning and embracing of change in practice from within including the identification of strategies to overcome difficulties and maximise benefits of implementation.

**Acknowledgements** This study is co-funded by the Irish Health Research Board Applied Partnership Award and the Irish Hospice Foundation

## O3 Improving European Healthcare Systems through the Development of a Realist Evaluation Framework for a European Public Health Data Analytic Project

### Andrew Boilson^1^, Regina Connolly^2^, Anthony Staines^1^, Paul Davis^2^, Justin Connolly^1^ , Dale Weston^3^

#### ^1^ School of Nursing and Human Sciences, Dublin City University, Glasnevin, Dublin 9, Ireland; ^2^ Business School, Dublin City University, Glasnevin, Dublin 9, Ireland; ^3^ Emergency Response Department, Science and Technology, Health Protection Directorate, Public Health England, Porton Down, Salisbury, UK

**Correspondence:** Andrew Boilson (andrew.boilson2@mail.dcu.ie)

**Background**

The multi-national MIDAS (Meaningful Integration of Data Analytics and Services) project is developing a big data platform to facilitate utilisation of a wide range of health and social care data to enable integration of heterogeneous data sources, providing analytics, forecasting tools and bespoke visualisations of actionable epidemiological data.

**Method**

An evaluation framework starting with a logic model and semi-structured interviews using the principles of realist evaluation was developed working with 6 developers, 5 health service managers, 2 allied health professionals and 3 policy makers. Parallel case studies were used to address the requirements of stakeholders at critical time points during the project.

**Results**

At this early stage of the MIDAS project’s development, the logic model represents an accurate representation of the project’s key outputs, outcomes and impacts identified through the logic model transcripts coding process. The logic model was developed through a number of iterations of consultation with the MIDAS consortium as an initial process of evaluating, planning and developing the project.

As the project progresses, key indicators (outputs, outcomes and impacts) that were not initially included in the logic model may be identified through interviews with end users of the MIDAS platform tools and the technical development teams.

**Conclusion**

The objective of this study is to narrow the gap between end user requirements of the platform tools and technical developers’ expectations of the end user needs, an ongoing process of refining the logic model is required at critical stages of the project’s development. To narrow the gap an ongoing process of refining the logic model is required at critical stages of the project’s development. Overall, the early stage interviews indicated the logic model is an effective framework for the evaluation of the project.

## O4 Scaling up with structure: Impact, feasibility and theoretical reflections on a practice-informed scale up template to improve patient safety

### Andrew Sibley, Tracy Broom

#### Wessex Academic Health Science Network, Innovation Centre, Southampton, UK.

**Correspondence:** Andrew Sibley (andrew.sibley@wessexahsn.net)

**Background**

In 2017/18 Wessex Patient Safety Collaborative (PSC) led a project to support local NHS teams expand established patient safety initiatives. The aim was to develop a ‘scale up template’ (SUT) and test its use in practice. The SUT undertook “deliberate efforts to increase the impact of innovation to benefit more people and to foster policy and program development on a lasting basis.” [1] The SUT aimed to support “scale up to at least 60% of a target population that could potentially benefit from the program.” [2] The SUT developed a 10-stage process to scale up and used pragmatic techniques of engagement, face-to-face, with staff to work through the complexities of scale up.

**Method**

A mixed methods investigation, of the impact and feasibility of the SUT, collected quantitative scale up data on each initiative and undertook qualitative focus groups with staff.

**Results**

Quantitative findings indicated all initiatives reached their target of 60% (55 of 92 possible) scaled up units (hospital wards, care homes etc). Sixteen themes from a synthesised thematic analysis of four focus groups were identified. Themes included: Overwhelmingly positive perceptions of SUT; SUT changed the previous scale up approach; Train the trainer potential. Facilitator themes included: Project manager role vital; First meeting with AHSN PSC lead vital; SUT developed staff awareness of implementation science/scale up; Executive sponsorship vital. A key barrier theme was the systemic underestimation, by staff, of scale up complexity and challenges.

**Conclusion**

The SUT was perceived as a positive force for change as teams pragmatically worked with complexity to scale up their initiatives. The SUT was a catalyst for changing approach after previous failed attempts to scale up had occurred. Active ingredients of its value were identified. Reflections on how the SUT mapped to implementation theories was undertaken to further develop the SUT.

**Acknowledgements**

On behalf of Wessex AHSN and Wessex Patient Safety Collaborative, we would like to thank the NHS staff who participated in this scale up initiative and evaluation.

**References**

1. Norton WE, McCannon CJ, Schall MW, Mittman BS. A stakeholder driven agenda for advancing the science and practice of scale up and spread in health. Implementation Science. 2012; 7: 118.

2. Rabin BA, Brownson RC. Developing the terminology for dissemination and implementation of research. Dissemination and implementation research in health. 2012; DOI:10.1093/acprof:oso/9780199751877.003.0002

## O5 The role of knowledge exchange in scaling-up a complex intervention for osteoarthritis (ESCAPE-pain) in England

### Andrew Walker^1,2^, Annette Boaz^2^, Michael V Hurley^1,2^

#### ^1^Health Innovation Network, London, UK; ^2^Kingston University and St George’s, University of London, London, UK

**Correspondence:** Andrew Walker (andrew.walker8@nhs.net)

**Background**

Implementing and scaling-up innovations is a collective social process [1,2]. However, there is limited analysis about how knowledge exchange operates in practice to support scaling-up [3]. ESCAPE-pain is an evidence-based complex intervention for osteoarthritis currently in >100 sites in England. The study investigated the role of knowledge exchange in scaling-up ESCAPE-pain.

**Method**

A qualitative case study approach was used. Forty clinicians and managers participated in 50 interviews across 14 organisations in England. Four NHS providers were selected as case studies, each comprising 5-7 participants. A fifth case study was undertaken of a clinical-academic network’s role in supporting the scale-up of ESCAPE-pain. This used an ethnographic approach comprising multiple interviews with project and senior managers, 8 months of observations and documentation.

**Results**

Two themes are explored.

1) Creating spaces to exchange formal and tacit knowledge:

- Physical spaces – large stakeholder knowledge sharing events and partnership working with early adopters were used to share learning about implementing ESCAPE-pain.
- Digital spaces – a website and app were developed to expand geographical reach and provide practical knowledge about ESCAPE-pain and its implementation.

2) Types of knowledge created and its purpose – the knowledge exchange process developed understanding about:

- ESCAPE-pain and how to manage osteoarthritis better through manualising it, developing a training course, and creating resources (e.g. return on investment tool).
- How to implement ESCAPE-pain to expand reach across different settings, widening target populations, and broadening the workforce delivering it (e.g. clinician/non-clinicians).

**Conclusion**

The study provides empirical evidence that builds on reframing the idea of the evidence-practice gap as one of a “space” of interaction [4]. The clinical-academic network actively facilitated better connectivity across systems to support the exchange and production of knowledge to drive spread. It was a non-linear and emergent process that utilised formal and tacit knowledge. This has implications for planning system-wide scale-up.

**Acknowledgements**

We would like to thank the participants who took part on the study. Health Innovation Network for funding the study.

**References**

1. Lanham HJ, Leykum LK, Taylor BS, McCannon CJ, Lindberg C, Lester RT. How complexity science can inform scale-up and spread in health care: understanding the role of self-organization in variation across local contexts. Soc Sci Med 1982. 2013 Sep; 93:194–202.

2. May CR, Johnson M, Finch T. Implementation, context and complexity. Implement Sci. 2016;11:141.

3. Ward V, Smith S, House A, Hamer S. Exploring knowledge exchange: a useful framework for practice and policy. Soc Sci Med 1982. 2012 Feb;74(3):297–304.

4. Kitson A, Brook A, Harvey G, Jordan Z, Marshall R, O’Shea R, et al. Using Complexity and Network Concepts to Inform Healthcare Knowledge Translation. Int J Health Policy Manag. 2018 Mar 1;7(3):231–43.

## O6 Assessing causal links and potential consequences of implementing innovations in complex systems – A worked example of applying participatory systems thinking methods in a regional emergency medical service system in Oldenburg, Germany

### Anja Sommer^1,2^, Cassandra Rehbock^1^, Thomas Krafft^1^, Alexandra Ziemann^1,3^

#### ^1^Faculty of Health, Medicine and Life Sciences, Care and Public Health Research Institute, Maastricht University, Maastricht, The Netherlands; ^2^Department of Anesthesiology, University Hospital Aachen, RWTH Aachen University, Aachen, Germany; ^3^CHIR Centre for Healthcare Innovation Research, City, University of London, London, United Kingdom

**Correspondence:** Anja Sommer (a.sommer@maastrichtuniversity.nl) & Cassandra Rehbock (c.rehbock@maastrichtuniversity.nl)

**Background**

We assessed the usefulness of systems thinking methods to guide the implementation of interventions reducing demand in a German emergency medical service (EMS) system with the aim of

- analysing intended and unintended consequences of implementing innovations in a complex system; and
- enhancing stakeholder involvement in the implementation process.

**Method**

Within a stakeholder group (EMS managers, frontline staff, health insurance representatives), causes and consequences of ‘rising EMS demand’ were discussed and incorporated into a causal loop diagram [1]. Potential interventions to reduce demand were identified by the group and incorporated into the diagram. A pathway analysis was conducted identifying intended or unintended consequences of prioritised interventions in the system [2]. Next steps include assessing time delays, a cross-comparison of the interventions’ impact and a stakeholder evaluation of the methodology.

**Results**

36 variables in four thematic groups were identified as interlinked causes/consequences of demand rise (staff, patients, other services, policy level). Eight feedback loops were identified. Four of 13 interventions were included in the path analysis showing no unintended consequences in the system (Figure 1). Results of the next analysis steps will be presented at the conference.

**Conclusion**

Applying systems thinking methods helped understanding the complex interactions in the EMS system around the specific problem of demand rise and assess the impact of potential interventions. The methodology allowed for the interactive involvement of the stakeholders from the beginning of the implementation process.

**Acknowledgements**

Both corresponding authors have contributed equally to this paper and count as first authors. We also thank the Oldenburg-project consortium for their steady input throughout the process of this work.

**References**

1. Schoenenberger LK, Bayer S, Ansah JP, Matchar DB, Mohanavalli RL, Lam SSW, Ong MEH. Emergency department crowding in Singapore: Insights from a systems thinking approach. SAGE Open Med. 2016; 4: 1 - 10.

2. Schoenenberger L, Schenker-Wicki A, Beck M. Analysing Terrorism from a Systems Thinking Perspective. Perspectives on Terrorism. 2014; 8: 16 - 36.

**Figure 1 (abstract P6).**
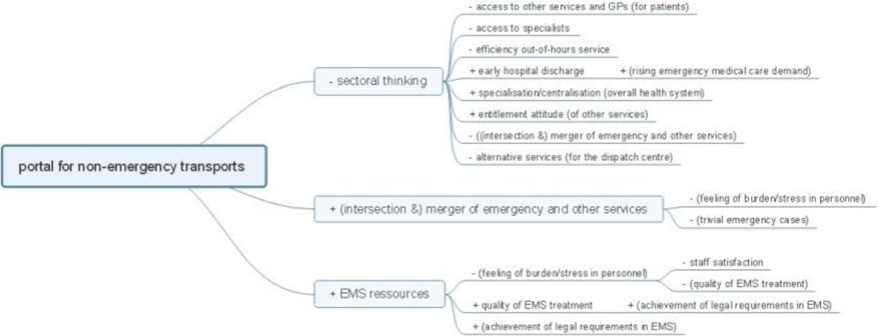
Extract of a pathway analysis for one intervention. (+ = reinforcing, - = balancing)

## P7 Can a short course increase the skills of early career researchers in initiating and sustaining partnerships with policy? Findings from a pilot evaluation

### Anna Williamson^1,2,3^, Hannah Tait^1^, Sian Rudge^1^, Louisa Jorm^2^, Sally Redman^1^

#### ^1^The Sax Institute, Sydney, NSW, Australia; ^2^The University of New South Wales, Sydney, NSW, Australia; ^3^ The University of Sydney, Sydney, NSW, Australia.

**Correspondence:** Anna Williamson (anna.williamson@saxinstitute.org.au)

**Background**

Researchers internationally are increasingly motivated to work in partnership with policy agencies on research projects. Such partnerships can be challenging to initiate and sustain, however, and there are few resources available to guide researchers wishing to build skills in this space. The current study presents pilot data from a short course designed to increase the capacity of early career researchers to initiate and sustain research partnerships with policy agencies. The specific aims of the study are to assess the: a) Uptake and acceptability of the course; b) Impact of the course on intentions to work with policy makers and confidence in doing so; and c) Extent to which the impacts (if any) of the course were sustained six months post attendance.

**Method**

A pilot evaluation of the first two rounds of this course, developed by the Centre for Informing Policy in Health with Evidence from Research (CIPHER) and the Sax Institute, was conducted. Process measures were collected to assess feasibility, a post-course feedback form was administered to assess acceptability. Participants completed a short questionnaire rating their skills and confidence in working with policy makers, pre, immediately post and six months post course attendance.

**Results**

The course was feasible to run. Ten participants attended and completed each course (as planned) and feedback data suggested the course was highly acceptable to participants (average ratings of ≥’satisfied’ for all items). Significant improvement in self-reported skills, confidence and intentions regarding working with policy agencies were noted amongst participants post course and almost all were maintained at six month follow up.

**Conclusion**

Early data suggests that this short course is a feasible and effective way of improving early career researchers self-reported skills, confidence and intentions to work in partnership with policy agencies on research projects.

**Acknowledgements**

This course was developed as part of the Centre for Informing Policy in Health with Evidence from Research (CIPHER), CIPHER was a National Health and Medical Research Council Centre for Research Excellence (APP1011436). The evaluation of this course was funded by a NSW Health Early-Mid Career Research Fellowship awarded to Dr Anna Williamson.

## O8 Implementing the children and young people’s IAPT quality improvement initiative in Cambridgeshire: findings from a process evaluation

### Anne-Marie Burn^1^, Maris Vainre^1^, Ayla Humphrey^2^, Emma Howarth^1^

#### ^1^ NIHR CLAHRC East of England, Institute of Public Health, University of Cambridge, Cambridge, UK; ^2^ Department of Psychiatry, University of Cambridge, Cambridge, UK

**Correspondence:** Anne-Marie Burn (amb278@medschl.cam.ac.uk)

**Background**

In 2011, the Children and Young People’s Improving Access to Psychological Therapies (CYP-IAPT) programme was introduced nationally to improve outcomes and experience of care for children and young people. The aim was to support services to embed key CYP-IAPT principles into everyday clinical practice so as to deliver evidence-based practice, demonstrate accountability through outcome monitoring, increase access to effective services and engage children and young people to participate in decisions about treatment and services. The current study explored the process of implementation and the experiences of professionals in specialist mental health settings in Cambridgeshire.

**Methods**

i) An audit and analysis of national and local documents issued between 2011 and 2015 and mapped along a five-year timeline; ii) Thematic analysis of in-depth interviews with 20 staff working in three specialist mental health settings in the Cambridgeshire and Peterborough Foundation Trust, conducted at two time points along the implementation trajectory.

**Results**

While there was high investment from frontline staff, there was a lack of clarity about the scope and aims of the CYP-IAPT programme during the early implementation phase. Programme developers emphasised some CYP-IAPT principles more than others and there was variation in perceptions of the extent to which principles were embedded. The creation of dedicated staff posts were a key driver to implementing and sustaining the CYP-IAPT model at a local level; specialist training and enhanced supervision facilitated evidence-based practice and outcome monitoring. Barriers to implementation included inadequate and inflexible IT systems, time limited funding and a lack of support from senior management. Organisational differences between partner agencies led to ineffectual collaborative working and high staff turnover prevented knowledge continuity.

**Conclusion**

This study provides valuable insights into local implementation of a complex national quality improvement programme. Recommendations will inform other local quality improvement initiatives relating to children and young people’s mental health.

## P9 Developing an implementation strategy for the use of objective adherence data in routine clinical practice: a case study in cystic fibrosis clinics

### Carla Girling^1^, Daniel Hind^1^, Madelynne A Arden^2^, Martin J Wildman^3^

#### ^1^Clinical Trials Research Unit, School of Health and Related Research, The University of Sheffield, Sheffield, UK; ^2^Department of Psychology, Sheffield Hallam University, Sheffield, UK; ^3^Sheffield Adult CF Centre, Sheffield Teaching Hospital NHS Foundation Trust, Sheffield, UK

**Correspondence:** Carla Girling (c.girling@sheffield.ac.uk)

**Background**

Preventative inhaled treatments preserve lung function and prolong life in Cystic Fibrosis (CF). An online platform (CFHealthHub) has been developed with patients and clinicians to support treatment habits. Self-report adherence to these treatments is over-estimated. CFHealthHub displays real-time objective adherence data from dose-counting nebulisers, so that clinical teams can offer informed treatment support.

In this paper, we used the theoretical domains framework (TDF) to identify implementation barriers to health professionals accessing objective adherence data through CFHealthHub. In 2019, scale up of implementation is required in a further 19 CF centres.

**Method**

Qualitative data were collected through semi-structured interviews with health professionals in three participating UK CF centres. The participants (n=13) were purposively sampled based on location and professional category. A topic guide was created exploring all theoretical domains. Transcripts were analysed by two researchers using framework analysis.

**Results**

Interviews demonstrated that participants did not have routine habits for using adherence data in clinical care. Analysis indicated that an implementation strategy should address all 14 domains to successfully support implementation scale up.

Participants reported insufficient training and low confidence in using adherence data. As a result, participants reported negative beliefs that adherence data would be used to “tell patients off”.

In addition, participants frequently justified lack of engagement because they believed adherence was primarily a physiotherapist responsibility.

Environmental barriers, such as computer access and pressures on staff time were common. Participants thought environmental and social influence barriers could be addressed by dedicated senior management.

**Conclusion**

The identified barriers supported the development of an implementation strategy using the behaviour change wheel. The strategy includes support for habit formation and other barriers using intervention functions, such as environmental restructuring, enablement, education and modelling. The success of this strategy will be evaluated as the project opens in new CF centres.

**Acknowledgements** CFHealthHub Data Observatory is funded by NHS England Commissioning for Quality and Innovation

**Trial Registration** ISRCTN14464661

## O10 What are the relationships between contexts, mechanisms and outcomes which underpin the ‘dynamic sustainability’ of a complex health care intervention across a system?

### Carrie-Ann Black, Nick Sevdalis, Crispin Day, Lucy Goulding

#### ^1^Centre for Implementation Science, Health Service Population Research, IOPPN, King’s College, London, UK

**Correspondence:** Carrie-Ann Black (carrie-ann.black@kcl.ac.uk)

**Background**

Whilst there is agreement within the implementation science arena that sustainability is a key outcome, limited attention has been given to understanding how to sustain complex interventions in practice [1]. Historically, the emphasis has been on evaluating and exploring the initial implementation, with time and budgetary constraints often meaning that longer term sustainability has not been a focus. To understand what affects the determinants of sustainability, we need to understand the relationships between determinants. To address this gap, we present a review of the evidence base which aims to contribute to our understanding of these determinants and how they interrelate in different contexts.

**Method**

A systematic rapid realist review of the literature was undertaken, to examine the relationships between contexts, mechanisms and outcomes which underpin the ‘dynamic sustainability’ of a complex health care intervention across a system. Evidence was submitted to thematic content analysis and synthesised to create a series of Context-Mechanism-Outcome Configurations (CMOCs). Testable hypotheses based on these configurations were derived for further research.

**Results**

539 studies were screened, of which 17 studies were included in the review. Projects were initiated in a variety of healthcare settings and represented a range of complex interventions. The findings include a series of 17 CMOCs each illustrating how, triggered by specific contextual factors a combination of programme resource and stakeholder reasoning led to specific outcomes.

**Conclusion**

The review identified for the first time a set of CMOCs that determine sustainability of complex healthcare interventions. These CMOCs are empirically testable. Our further research focuses on studying how these CMOCs impact on sustainability outcomes.

**References**

1. Wiltsey Stirman, S., Kimberly, J., Cook, N., Calloway, A., Castro, F. and Charns, M. The sustainability of new programs and innovations: a review of the empirical literature and recommendations for future research.IS. 2012; 7 (17). Available from https://doi.org/10.1186/1748-5908-7-17

## P11 Implementation of non-specialist delivered counselling for depression and harmful alcohol use in South Africa: managers’ perspectives

### Carrie Brooke-Sumner^1, 2^, Petal Petersen-Williams^1,2^, James Kruger^3^, Hassan Mahomed^3,4^, Bronwyn Myers^1,2^

#### ^1^Alcohol, Tobacco and Other Drug Research Unit, South African Medical Research Council, Francie Van Zijl Drive, Parow Valley, Cape Town 7501, South Africa; ^2^Department of Psychiatry and Mental Health, University of Cape Town, J-Block, Groote Schuur Hospital, Observatory, Cape Town, South Africa; ^3^Western Cape Government: Health, Norton Rose House, 8 Riebeeck Street, Cape Town 8001, South Africa; ^4^Division of Health Systems and Public Health, Department of Global Health, Faculty of Health Sciences, Stellenbosch University, Francie van Zijl Drive, Tygerberg, Cape Town 7505, South Africa

**Correspondence:** Carrie Brooke-Sumner (Carrie.Brooke-Sumner@mrc.ac.za)

**Background**

Primary Health Care (PHC) Facility Managers have a key role in promoting and sustaining implementation of health innovations. The Project MIND randomised controlled trial is evaluating integration of counselling for depression and harmful alcohol use into current chronic disease care. This qualitative study investigated the experiences of Facility Managers who delivered the MIND counselling during the trial.

**Method**

This counselling has been implemented in 16 PHC facilities. We aimed to conduct three focus group discussions with the managers from these sites. Two focus groups were conducted; however, participation was low (n=7 total). We followed up with in-depth individual interviews with 8 Facility managers and 1 Operational manager. Interviews were conducted in English and transcribed verbatim. Thematic analysis in NVivo 12 was conducted by two researchers independently.

**Results**

Three main themes emerged.

1. **Perceived benefits underpin managers’ support for implementation.** All managers were supportive of the wider implementation of the counselling. They described a range of benefits including (i) prioritisation of mental disorders in the facility (ii) addressing social determinants not addressed by clinical care.
2. **Improving facility communication.** Most managers felt implementation would benefit from additional and ongoing communication (e.g. in staff meetings) about the counselling, due to constant turnover of staff and complexity of the intervention.
3. **Strategies for improving integration into current services.** These included (i) linking with mental health nurses for counsellors to attend to less severe cases (ii) ensuring feedback to the treating clinician (e.g. HIV treatment) regarding outcome of counselling.

**Conclusion**

Facility managers in South Africa are a difficult to reach group given the pace and nature of their work [1]. This study indicates their support for implementation of MIND counselling despite health system constraints. Participants focused on facilitators to enable the counselling to be integrated into their facilities, which should be incorporated into future implementation studies.

**Reference**

1. Brooke-Sumner C, Petersen-Williams P, Kruger J, Mahomed H, Myers B. ‘Doing more with less’: a qualitative investigation of perceptions of South African health service managers on implementation of health innovations. Health Policy and Planning. 2019; 34: 132-140

## O12 Developing products for knowledge mobilisation of healthcare research

### Charlotte A Sharp ^1, 2, 3^, William G Dixon ^4^, Ruth Boaden^1, 3^, Caroline Sanders^2^

#### ^1^National Institute for Health Research Collaboration for Leadership in Applied Health Research and Care, Greater Manchester, Salford Royal NHS Foundation Trust, Salford, UK; ^2^National Institute for Health Research School for Primary Care Research, The University of Manchester, Williamson Building, Oxford Road, Manchester, UK; ^3^Alliance Manchester Business School, The University of Manchester, Manchester, UK; ^4^Arthritis Research UK Centre for Epidemiology, Division of Musculoskeletal and Dermatological Sciences, School of Biological Sciences, The University of Manchester, Manchester, UK

**Correspondence:** Charlotte A Sharp (Charlotte.sharp@manchester.ac.uk)

**Background**

The current emphasis on demonstrating academic ‘impact’ contributes to the proliferation of knowledge mobilisation (KM) products from healthcare research (e.g. toolkits, guidance etc), which may be referred to as ‘boundary objects’. A research gap exists regarding the motivation for their development, development processes and the factors influencing whether and how they mobilise knowledge.

**Methods**

Phase 1: Semi-structured interviews (20) + focus group (11) (academics, healthcare managers, funders) focussing on perceptions of toolkits. Phase 2: Four case studies of applied healthcare research projects developing KM products. An ethnographic approach generated data from observations (80+ hours), document analysis (>150) and interviews (40). Thematic analysis was inductive and deductive, building on Phase 1 themes and developing new themes where appropriate.

**Results**

Phase 1 saw the academic context constraining researchers’ ability to concentrate on developing KM products. Toolkits were perceived as ill-defined, practical resources, helping users put knowledge into practice. Participants reported overwhelming cynicism towards them yet felt obliged to produce them. Phase 1 themes supported Phase 2, where the impact agenda perpetuates KM product development as stand-alone interventions instead of a broader KM strategy. Cross case analysis revealed themes influencing the potential to move KM products from ‘designated boundary objects’ to ‘boundary objects in use’: need (ranging from unclear to identified gap), audience (ill-defined to clear), team dynamic (dysfunctional to collaborative), leadership ((dis)engaged), project management (chaotic to clear) stakeholder engagement (tokenism to co-production), and product (ill-defined to boundary object-in-use). KM products were characterised as symbolic boundary objects, used as bargaining chips with funders or research subjects.

**Conclusion**

To optimise the potential application of KM products, researchers and funders should consider the motivation for their development. Where developing such a product is felt, potentially, to make a meaningful impact in practice, carefully planning stakeholder engagement, collaboration and project management might enhance their impact.

**Acknowledgements**

Charlotte A Sharp is supported by the National Institute for Health Research Collaboration for Leadership in Applied Health Research and Care (NIHR CLAHRC) Greater Manchester. The views expressed are those of the authors and not necessarily those of the NIHR, the NHS or the Department of Health and Social Care.

## O13 Of distribution and drift, the role of everyday technologies in the implementation of care bundles

### Charlotte Overton

#### Centre for Health Innovation Leadership and Learning, Nottingham University Business School, University of Nottingham, UK

**Correspondence:** Charlotte Overton (charlotte.overton@nottingham.ac.uk)

**Background**

In the NHS there are challenges regarding the implementation, sustainability and spread of quality improvement innovations [1]. Technology in practice offers a useful theoretical perspective to explore the implementation of quality-improvement tools. By applying this perspective, tools are viewed as everyday technologies that play an active part in healthcare as opposed to being inanimate objects in the form of paper- based or computer-based tools [2, 3].

**Method**

This study sought to systematically and critically examine the role quality-improvement tools play in healthcare organisations. Using sepsis as an example, the study explored two comparative case studies of implementation strategies used to improve the recognition and treatment of sepsis. Ethnographic observation in the Emergency Department (ED), ward settings and relevant meetings (200 hours), semi-structured interviews (60) and documentary analysis were used. The data were analysed using thematic analysis.

**Results**

The empirical findings showed that when implemented there was a gap between tools and practice which was crossed by a chain of re-representations. Each re-representation contained a multitude of actions and decisions that were; highly distributed across a range of heterogeneous entities such as the tool itself, healthcare workers and equipment. This resulted in workarounds and various elements drifting from the original intention of the tools. For example, using the tool in the ED was different compared to the acute inpatient setting. In the ED work practices were directed towards the recognition and treatment of acute illness. Conversely, in acute inpatient settings work practices were directed towards a multitude of other activities.

**Conclusion**

This research provides valuable insights into where distribution and drift occur. The findings contribute towards an understanding of the mechanisms by which improvement programmes work. An understanding of the role that tools play when used in practice can lead to more efficient and effective implementation, sustainability and spread of quality improvement innovations.

**Acknowledgements** Professors Justin Waring and Stephen Timmons and Dr Emma Rowley for their supervision of this PhD.

**References**

1. Horton T., Illingworth J., Warburton W. The spread challenge. The Health Foundation. 2018

2. Berg M. Of Forms, Containers and the Electronic Medical Record: some tools for a sociology of the formal. Science Technology and Human Values. 1998; 22: 403-433.

3. Allen D. From polyformacy to formacology. BMJ Quality and Safety. 2017. 26: 695-697.

## P14 Implementation Science Research Development (ImpRes) Tool Protocol Assessment Criteria (ImpResPAC): Development and Evaluation

### Chloe Sweetnam, Lucy Goulding, Louise Hull

#### Centre for Implementation Science, Department of Health Service and Population Research, Institute of Psychiatry, Psychology and Neuroscience, King’s College London, United Kingdom

**Correspondence:** Lucy Goulding (lucy.goulding@kcl.ac.uk) & Louise Hull (louise.hull@kcl.ac.uk)

**Background**

The **Imp**lementation science **Res**earch development (**ImpRes**) tool and supplementary guide have been developed to provide guidelines on how to design high-quality implementation science research [1]. ImpRes did not include quantitative assessment criteria for evaluating the quality of implementation science protocols: these were developed in this study to identify strengths and weaknesses in implementation science research protocols and make recommendations to address current deficits in implementation research design.

**Method**

This study developed ‘**Imp**lementation science **Res**earch development tool **P**rotocol **A**ssessment **C**riteria’ (ImpResPAC), a protocol scoring system, to quantitatively evaluate implementation science protocols for information provided on five out of the 10 ImpRes domains: research characteristics, implementation theory frameworks and models, implementation strategies, implementation outcomes, and unintended consequences. ImpResPAC was applied to 16 implementation science protocols, published in Implementation Science, to identify strengths and weaknesses of planned implementation science research.

**Results**

The majority of protocols scored highly on describing the research gap, but few differentiated between the implementation activities associated with each implementation stage. Although most protocols provided strong rationale for the choice of theory, framework and model underpinning the research, many lacked thorough application throughout the implementation research. In most cases, the implementation strategy was explicitly described and justified. Many protocols described the intention to measure fidelity, but few intended to measure appropriateness, acceptability, penetration and sustainability. Only two of the protocols intended to measure unintended consequences. The reliability of ImpResPAC was evaluated by conducting intra-class correlation coefficient (ICC) tests on a subset of protocols and demonstrated agreement of 0.85 (ICC).

**Conclusion**

The application of ImpResPAC to 16 protocols identified strengths and weaknesses in research design. Furthermore, ImpResPAC presents a reliable scoring system that researchers, funders and decision-makers can use as a learning tool to self-assess implementation science research protocol quality.

**Reference**

1. King’s Improvement Science, *Implementation Science Research Development (ImpRes) tool and guide*. Available from: http://www.kingsimprovementscience.org/ImpRes [accessed 1st April 2019].

## P15 Scaling up a polypharmacy Action Learning Sets tool: interim findings and methodological insights

### Cindy F. Brooks^1^, Anastasios Argyropoulos^1^, Catherine B. Matheson-Monnet^1^, Richard Guerrero-Luduena^1^, David Kryl^1,2^, Ruth George^2^, Vicki Rowse^2^ and Clare Howard^2^

#### ^1^ Centre for Implementation Science, University of Southampton, Southampton, UK; ^2^ Wessex Academic Health Science Network, Southampton, UK

**Correspondence:** Cindy F. Brooks (C.F.Brooks@soton.ac.uk)

**Background**

In line with national policy, to address the issues associated with inappropriate polypharmacy, the Centre for Implementation Science are evaluating the implementation of a Wessex Academic Health Science Network led Action Learning Sets (ALS) tool, to improve healthcare practitioners’ confidence, perceptions and experiences of stopping inappropriate medicines safely.

**Methods**

Up to 125 healthcare practitioners attending ALS across three localities in the South of England are being invited to participate in the study. A mixed method approach involving pre-post ALS questionnaires, evaluation forms and observational methods, will be used to evaluate the study.

To date, interim findings have been analysed from one locality (n=33), and methodological insights recorded which relate to the prospective scale up and evaluation of the ALS across two further localities. Quantitative paired pre-post ALS questionnaire responses were analysed using the Wilcoxon signed-rank test, whilst qualitative data were analysed using thematic analysis and ethnographic methods. Overall impact of the ALS will be evaluated using Kirkpatrick’s evaluation model.

**Results**

Interim findings show the ALS has contributed to improving perceptions, confidence and experiences in addressing polypharmacy and more specifically perceptions of; stopping inappropriate medicines, use of knowledge, information and tools, shared decision making with patients/clinicians and the role of institutional factors. Methodological insights relating to the scale-up of the ALS across two further localities include the impact of locality upon participation, as well as the process of engagement of participants in the evaluation.

**Conclusion**

Interim findings and methodological insights in scaling up the study may be of relevance to national policy to understand how to address polypharmacy issues in different localities.

## P16 The Life Link Clinic: Meeting the socio-economic needs of patients and families within complex rehabilitation in-patient services

### Claire Hendry^1,2,3^, Clarissa Giebel^3,4^, Tim Villanueva^1,2,5^, Nicola Hill^1,6^, Thomas Spearitt^1,2^, Lea Johnson^1,5^, Clare Moore^1,2^, Jon Smith^1,2,5,6^, Siobhan Mealey^7^, Peter Bailey^3^, Jennifer Bailey^3^, Heather Burnage^1,2^, Michelle Hughes^1,2,6^, Maria Moyo^1,2^

#### ^1^The Cheshire and Merseyside Rehabilitation Network, The Walton Centre NHS Foundation Trust, Liverpool, UK; ^2^ The Walton Centre NHS Foundation Trust, Liverpool, UK; ^3^ NIHR CLAHRC North West Coast, Institute of Population Health Sciences, University of Liverpool, Liverpool, UK; ^4^ Institute of Population Health Sciences, University of Liverpool; Liverpool, UK; ^5^ The Royal Liverpool and Broadgreen University Hospitals NHS Trust, Liverpool, UK; ^6^St Helens and Knowsley NHS Trust, Merseyside, UK; ^7^ The Brain Charity, Liverpool, UK

**Correspondence:** Claire Hendry (Claire.hendry@thewaltoncentre.nhs.uk)

**Background**

Feedback from patients and families in complex rehabilitation services highlighted that socio-economic issues increased their stress and anxiety levels. In response, an integrated provision is being piloted within complex rehabilitation inpatient settings under the umbrella of the Cheshire and Merseyside rehabilitation network. This service aims to offer a multi-agency approach to support patients with any socioeconomic issues, to help reduce stress and anxiety, to promote integrated accessible support.

**Method**

We conducted sixteen Semi-structured interviews with staff and service users to evaluate the existing three services and improve the implementation of the two new sites. Using the DASS 21 and the NHS family and friends test, service users are asked to provide feedback on the service and rate their stress and anxiety before and after accessing the service. Staff and service users’ perception of the service is also collected through semi-structured interviews. Using a mixed method analysis, the semi-structured interviews were thematically analysed and paired t-test utilised to compare changes in pre- and post- data.

**Results**

Interviews indicated that this support was considered essential, which helped to improve the two new sites implemented since December 2018. The service evaluation is on-going. Preliminary analysis suggests that stress levels in families are above the ranges which we see in patients on admission to the acute rehabilitation setting. Using information from the staff questionnaire, socio-economic issues encountered by patients, carers, and families were mapping across the main themes raised when accessing the service. This reflected that the service was meeting the needs of patients and families.

**Conclusion**

Findings from the evaluation of this implementation of two new sites and five in total across the North West Coast region can help to identify the benefits of this service to people with complex brain injuries and their families in terms of their social needs.

## O17 Evaluation of implementation of high-throughput Non-Invasive Prenatal Testing (NIPT) for foetal *RHD* genotype testing: results of a survey of maternity units in England, supplemented by expert elicitation findings

### Edyta Ryczek^1^, Judith White^1^, Grace Carolan-Rees^1^

#### ^1^Cedar, Cardiff and Vale University Health Board, Cardiff, United Kingdom

**Correspondence:** Edyta Ryczek (edyta.ryczek@wales.nhs.uk)

**Background**

An RhD-negative mother carrying an RhD-positive foetus is at high risk of sensitisation which can be reduced by administration of Routine Antenatal Anti-D Prophylaxis (RAADP). However, approximately 33% of women carry an RhD-negative baby and do not need RAADP [1]. In 2016, The National Institute for Health and Care Excellence recommended Non-Invasive Prenatal Testing (NIPT) for foetal *RHD* genotype as a cost-effective option to guide RAADP in the United Kingdom and established research recommendations investigated in this study^1^. The aim of the study was to evaluate the implementation of high-throughput NIPT for foetal *RHD* genotype in maternity units in England, the United Kingdom.

**Method**

A survey evaluating the implementation, antenatal care, uptake of the new test, RAADP adherence and postpartum care was sent to 39 Hospital Trusts in England. Topics which required further investigation were explored during qualitative interviews with seven clinicians chosen based on their geographical location. Test results from all hospitals in the United Kingdom which offer the new testing strategy are included.

**Results**

Nurses and midwives were most likely to have received training organised internally within a Trust. The new service is not expected to incur extra costs. Cost savings include reduction in Anti-D use, Anti-D quantification, and samples testing (cord blood typing, the Kleihauer test). RAADP appointments are shorter or not required which balances the extra time needed during the initial appointments. The frequency of positive, negative and inconclusive results is 55.9%, 34.5% and 4.3%, respectively. Neither the uptake of NIPT for foetal *RHD* genotype nor adherence to RAADP is routinely monitored. Trusts described using four different postpartum testing strategies.

**Conclusion**

NIPT for foetal *RHD* genotype fits well within the existing patient pathway. The success of implementation of the new testing was associated with good working relationship between staff and organisation of work within the department.

**Acknowledgements**

The authors would like to acknowledge Dr James Evans, Ruth Poole, Kathleen Withers, Dr Laura Knight and Dr Helen Morgan for their contribution to the study design, management and/or revision of the paper.

**References**

1. National Institute for Health and Care Excellence. High-throughput non-invasive prenatal testing for foetal RHD genotype. NICE diagnostic guidance 25; 2016.

## O18 Developing a scaling up plan for quality improvement training in urological surgery

### Elena Pallari^1,2^, Zarnie Khadjesari^1,3^, James Green^4^ and Nick Sevdalis^1^

#### ^1^ King's College London, Institute of Psychiatry, Psychology & Neuroscience (IoPPN), Centre for Implementation Science (CIS), Health Service and Population Research Department, London, UK; ^2^ MRC Clinical Trials and Methodology, University College London, London, UK; ^3^ School of Health Sciences, University of East Anglia, Norwich Research Park, Norwich, UK; ^4^ Bart’s NHS Trust, Whipps Cross Hospital, Urological Department, London, UK

**Correspondence:** Elena Pallari (elena.pallari@kcl.ac.uk)

**Background**

The education in quality improvement programme (EQUIP) for urology aims to address the need for an evidence-based curriculum in quality improvement (QI) methods training in surgery. Key goal of EQUIP is to be nationally scalable in the UK. Here we present the development of the EQUIP scale-up strategy.

**Methods**

The strategy was developed using evidence triangulation from mixed-methods studies: evidence-based curriculum development (evidence reviews), national gap analysis (qualitative data), and programme piloting at two UK sites for feasibility and effectiveness (quantitative data). The collected data involved a range of stakeholders: training programme directors and trainers as well urology trainees, and a steering group which included educational practitioners, patient representatives, trainees and consultant urologists. Further, we framed the implementation model of the scale up plan based on the Exploration, Preparation, Implementation, Sustainment (EPIS) framework (Fig 1).

**Results**

We identified prioritization challenges, Trust resources, and time commitments as the greatest barriers towards the scale-up of EQUIP, while structured approaches to QI work as the drivers towards developing an educational platform in QI techniques, a network to support undertaking QI work and creating a QI infrastructure.

**Conclusion**

This study offers an empirical and theoretical understanding of how to scale-up the EQUIP programme nationally in the UK. Based on the study, the scale-up strategy involves national stakeholder support; an IT platform; nationally shared training materials; and a train-the-trainers programme. The scale-up strategy will be evaluated further in the coming 2 years of EQUIP implementation and will be used to guide potential diffusion across other specialities.

**Acknowledgements**

The authors would like to thank the Training Programme Directors of each region, the trainers on the course and the Urology Surgery trainees who took part in the study.

**Funding**

This research work was supported from funding by The Urology Foundation and The Schroders Foundation. EP/NS are supported by the National Institute for Health Research (NIHR) Collaboration for Leadership in Applied Health Research and Care South London at King’s College Hospital NHS Foundation Trust. The views expressed are those of the authors and not necessarily those of the NHS, the NIHR or the Department of Health.

**Figure 1 (abstract 018).**
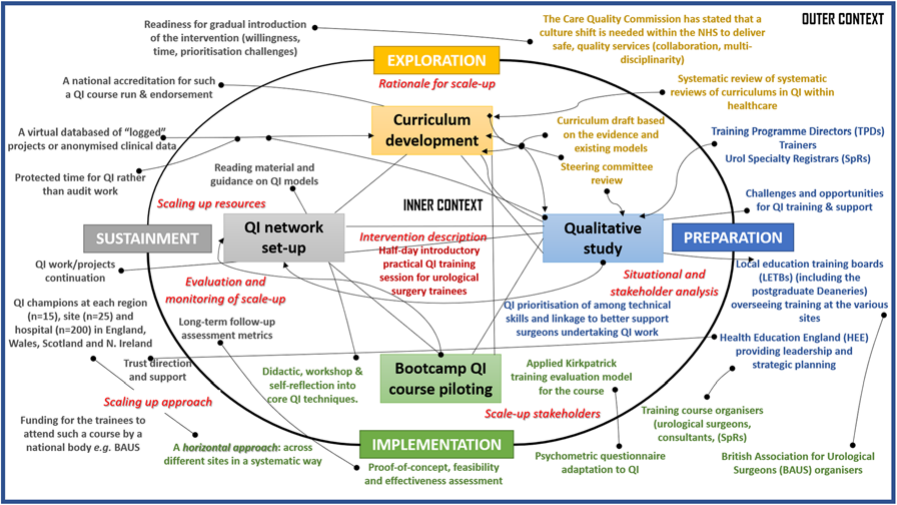
Empirical model of the scale-up plan using the EPIS framework and the conceptual phases and factors affecting implementation in QI training for urological surgery

## O19 The development and application of a logic model to translate an established Perioperative medicine for Older People undergoing Surgery (POPS) service to a novel setting

### Emily Jasper^1^, Nick Sevdalis^2^, R de las Casas^1^, C Meilak^3^, A Whittle^3^, Judith SL Partridge^1^, Jugdeep K Dhesi^1^

#### ^1^ Perioperative medicine for Older People undergoing Surgery (POPS) Service, Guy’s St Thomas Trust NHS, London, UK; ^2^ Centre for Implementation Science, Kings College London, London, UK; ^3^ Perioperative medicine for Older People undergoing Surgery (POPS), Dartford and Gravesham NHS Trust, Kent, UK

**Correspondence:** Emily Jasper (emily.jasper@gstt.nhs.uk)

**Background**

Older people are at higher risk of adverse perioperative outcome in comparison to younger patients and the need for geriatrician involvement in surgical pathways is advocated. An evidenced-based method of improving care for older surgical patients has been developed and embedded at Guy’s & St Thomas Trust (GSTT) and is being replicated nationally [1-5]. There is a call for scale up of Perioperative medicine for Older People undergoing Surgery (POPS) services but this requires a systematic approach [6]. A logic model is a critical initiating step to begin the scale up process with fidelity [7,8].

**Method**

Thirteen clinical and safety implementation experts were recruited to participate in three sessions to develop the POPS logic model. Guided by literature review and consensus, the model was constructed through mapping themes against core components of the Kellogg Logic Model Guide [8]. The model was applied and evaluated at Darent Valley Hospital (POPS@DVH).

**Results**

The logic model was used to design, develop, iterate and embed POPS@DVH. Over 18 months the new service resulted in improvements in process measures reductions in length of stay (-2.58 days) and readmission rates (-12%). Other key outcomes included improved documentation of medical complications (eg. improved delirium diagnosis from 0 to 21%) and comorbidities, translating to improved patient care and hospital coding. Stakeholder engagement was actively sought throughout this process, with high satisfaction noted from surgical teams, junior doctors and patients. The overall success of POPS@DVH was reflected in substantive surgical directorate funding allowing sustainability.

**Conclusion**

This study demonstrates the clinical utility of a logic model in facilitating successful scale up of an established POPS service into a novel setting. Further work would involve refinement of this logic model to establish a relevant and sustainable framework for a wider POPS scale up.

**References**

1. Ellis G, Whitehead M, O’Neill D, Langhorne P, Robinson D. Comprehensive geriatric assessment for older adults admitted to hospital (Review). Cochrane Library. 2011; 7.

2. Braude P, Goodman A, Elias T, Babic-Illman G, Challacombe B, Harari D, Dhesi J. Evaluation and establishment of a ward-based geriatric liaison service for older urological surgical patients: POPS-Urology (Proactive Care of Older People Undergoing Surgery). BJU International. 2016; 120:123-129.

3. Partridge J, Harari D, Martin F, Peacock J, Bell R, Mohammed A, Dhesi J. Randomised clinical trial of comprehensive geriatric assessment and optimisation in vascular surgery. BJS Society. 2017; 104:679-98.

4. Vilches-Moraga A, Fox J. Geriatricians and the older emergency general surgical patient: proactive assessment and patient centred interventions - Salford-POP-GS. Aging Clinical and Experimental research. 2018; 30:277-282.

5. Joughin A, Partridge J, O’Halloran T, Dhesi J. Where are we now in perioperative medicine? Results from a repeated UK survey of geriatric medicine delivered services for older people. Age and Aging; 2019; 0:1-4

6. Horton T, Illingworth J, Warburton W. The Spread Challenge – How to support the successful uptake of innovations and improvements in health care [Internet]. 2018 [Cited 25th Feb 2019]. Available from: https://www.health.org.uk/publications/the-spread-challenge

7. Medical Research Council. Developing and evaluating complex interventions [Internet]. 2019 [cited 28^th^ April 2019]. Available from: www.mrc.ac.uk/complexinterventionsguidance

8. W.K. Kellogg Foundation. Logic Model Development Guide [Internet]. 2004 [cited 22 February 2019]. Available from: https://www.wkkf.org/resource-directory/resource/2006/02/wk-kellogg-foundation-logic-model-development-guide

## O20 A systematic review and synthesis of facilitators and barriers to the implementation of evidence-based practices to support physiological labour and birth in obstetric settings

### Florence Darling, Christine McCourt and Martin Cartwright

#### City, University of London, London, United Kingdom

**Correspondence:** Florence Darling (florence.darling@city.ac.uk)

**Background**

One of the biggest challenges facing health-care professionals who care for women in labour and birth are decisions about the appropriate use of clinical interventions. Interventions for example caesarean-sections or instrumental births are necessary when problems arise, however routine use increases mortality and morbidity. We undertook a systematic review of studies to explore facilitators and barriers to the implementation of evidence-based practices to support physiological labour and birth, an important initiative, to reduce routine intervention use. We reviewed studies that explored practices in obstetric setting where routine intervention use is higher compared to midwife-led settings.

**Method**

Using PRISMA guidelines [1], databases was searched from 1990 to September 2018 and 31 original studies were included for thematic synthesis [2]. Analytic themes that were theoretically informed enabled us to explore facilitators and barrier at a micro level (obstetricians, midwives and women) and meso level (organisation) to implementing EBPs to support physiological labour and birth.

**Results**

The synthesis showed that prevalent risk perceptions of birth are an important barrier. This informed an approach based on risk surveillance and active management of labour. Obstetricians who hold strong risk perceptions of birth exert control over other professionals to apply a risk-based approach. An important barrier is their reluctance to relinquish this power. Approaches cognisant with EBPs to support physiological labour and birth is muted. Midwifery acquiesces, obstetric and midwifery preoccupation with risk surveillance and rationalisation of intervention use are important barriers. Women expect interventions to shape birth experiences. Centralisation of labour care sustains a risk-based approach. Facilitators included collaborative working by obstetricians and midwives to implement evidence-based practices, midwifery involvement in decision-making and organisational efforts to enhance midwifery autonomy

**Conclusion**

Future research should explore obstetrician’s reluctance to relinquish power, factors that facilitate collaborative working between professional groups, organisational influences and women’s experiences in obstetric settings.

Registration: International Prospective Register of Systematic Reviews (Ref: CRD42017081891)

**References**

1. Shamseer L, Moher D, Clarke M, Ghersi D, Liberati A, Petticrew M, et al. Preferred reporting items for systematic review and meta-analysis protocols (PRISMA-P) 2015: elaboration and explanation. BMJ: British Medical Journal. 2015;349(jan02): g7647.

2. Thomas J, Harden A. Methods for the thematic synthesis of qualitative research in systematic reviews. BMC medical research methodology 2008;8 (1):45.

## P21 Evaluation of a Physical Health Plan for people with psychosis: Protocol for a Quality Improvement study

### Julie Williams^1^, Nick Sevdalis^2^, Fiona Gaughran^3,4^

#### ^1^ Health Service and Population Research Department, Institute of Psychiatry, Psychology and Neuroscience, King’s College London, London , UK; ^2^ Centre for Implementation Science, Institute of Psychiatry, Psychology and Neuroscience, King’s College London, London, UK; ^3^ Psychosis Studies, Institute of Psychiatry, Psychology and Neuroscience, King’s College London, London, UK; ^4^ National Psychosis Service, South London and Maudsley NHS Foundation Trust, London, UK.

**Correspondence:** Julie Williams (julie.williams@kcl.ac.uk)

**Background**

People with serious mental illness (SMI) have poorer physical health compared to the general population. Reasons for this are complex but include the identification and treatment of physical health conditions. Creating tools to support people with SMI to assume more control of their physical health may help to ameliorate some of these problems. This study evaluates the use of a service user-held Physical health plan (PHP) for service users to determine whether its use increases uptake of physical health services.

Method

We have undertaken a pilot quality improvement study to test the use of the PHP [1]. This included testing a Theory of Change using focus groups with service users and staff and evaluating the use of the PHP in two community mental health teams using the RE-AIM implementation framework to guide the evaluation using qualitative and quantitative measures to do this (eg collecting data on how many people used the PHP, if they required support, if there was a change to uptake in physical health services, measuring Patient Activation)

**Results**

Focus groups were run with staff and service users before the pilot started which led to some change to the PHP and how it was used. 27 service users have completed the PHP. Participants have found it easy to use and were able to identify physical health needs. These were shared with clinical staff. Follow-up is ongoing which will include interviews with service users and clinical staff to elicit their views on using the PHP. Analysis of the implementation outcomes using the RE-aim framework is ongoing.

**Conclusion**

This study used an implementation framework to test a novel intervention for people with SMI. Participants found the PHP easy to use and analysis will help us to evaluate the success of the intervention and whether a larger-scale definitive RCT is warranted.

**Acknowledgements**

Thank you to all the service users, carers and academic and clinical staff who took part in the initial stages of the development of the PHP. Special thanks to the two service users who ran the focus groups.

This study/project is supported by the Maudsley Charity and the National Institute for Health Research (NIHR) Collaboration for Leadership in Applied Health Research and Care South London (NIHR CLAHRC South London) at King’s College Hospital NHS Foundation Trust. The views expressed are those of the author(s) and not necessarily those of the NIHR or the Department of Health and Social Care.

**Trial Registration**

ClinicalTrials.gov Identifier: NCT03178279. Registered date: 05/06/2017

**Reference**

1. Williams, J. Sevdalis, N. Gaughran, F. Evaluation of a Physical health plan for people with psychosis: a protocol for a quality improvement study. Pilot and Feasibility Studies. 2019, 5.8.

## P22 Measuring success of quality improvement in a mental health NHS Foundation Trust

### Kia-Chong Chua^1, 2^, Barbara Grey^2^, Michael Holland^2^, Claire Henderson^1^, Nick Sevdalis^1^

#### ^1^Institute of Psychiatry, Psychology & Neuroscience, King’s College London, SE5 8AF, UK; ^2^South London & Maudsley NHS Foundation Trust, SE5 8AZ, UK

**Correspondence:** Kia-Chong Chua (kia-chong.chua@kcl.ac.uk)

**Background**

Quality Improvement (QI) in healthcare is widespread [1] with diverse projects and target outcomes [2]. Success rates are highly variable, often with disappointing results [3]. Existing guidance on QI evaluation [4] does not aid organisational reporting on the impact of QI [5]. We aim to develop a framework for measuring success of QI to aid organisational learning and shed light on capacity building needs and resource allocation priorities in South London and Maudsley (SLaM) NHS Foundation Trust.

**Method**

A scoping exercise was conducted by an academic faculty (KC) who is embedded as a researcher-in-residence in SLaM. With combined inputs from QI team in SLaM and academic colleagues in Centre for Implementation Science as well as a rapid evidence scan, we designed a survey for retrospective summative assessment. A generic input-process-output framework was used for the survey to overcome the difficulty of making comparisons when project-specific aims and outcomes were heterogeneous and highly localised. Each QI team member then identified 3-5 “successful” and “unsuccessful” QI projects to create a gradient of contrast for small but rapid gains in learning about pertinent factors of QI success. We did not formally operationalise the definition of success in the survey so as to understand “work-as-done” rather than imposing a “work-as-imagined” criteria in the absence of an established definition of QI success [6]. Logistic regression was used to compare the impact of contextual, input and process factors on whether projects achieved their aims.

**Results**

Among 52 QI projects across five boroughs of London, 12 achieved their aims. Contextual and input factors showed very little impact. In contrast, process factors like stakeholder engagement and measurement plan showed sizable impact on whether projects achieved their aims.

**Conclusion**

Measurement plan and data quality matters more than contextual and input factors in determining success of QI.

**References**

1. Kaplan HC, Provost LP, Froehle CM, Margolis PA: The Model for Understanding Success in Quality (MUSIQ): building a theory of context in healthcare quality improvement. *BMJ Qual Saf* 2012, 21(1):13-20.

2. Chassin MR, Loeb JM: The ongoing quality improvement journey: next stop, high reliability. *Health Aff (Millwood)* 2011, 30(4):559-568.

3. Dixon-Woods M, Martin GP: Does quality improvement improve quality? *Future Hospital Journal* 2016, 3(3):191-194.

4. Parry G, Coly A, Goldmann D, Rowe AK, Chattu V, Logiudice D, Rabrenovic M, Nambiar B: Practical recommendations for the evaluation of improvement initiatives. *Int J Qual Health Care* 2018, 30(suppl_1):29-36.

5. Morganti KG, Lovejoy S, Haviland AM, Haas AC, Farley DO: Measuring success for health care quality improvement interventions. *Med Care* 2012, 50(12):1086-1092.

6. Hollnagel E: Why is work-as-imagined different from work-as-done? In: *Resilient Health Care. Volume 2*, edn. Edited by Wears RL, Hollnagel E. London: CRC Press; 2017.

## P23 Provider training and implementation of newborn care interventions at facilities in Malawi: a secondary analysis of Service Provision Assessment survey data

### Kimberly Peven^1^, Debra Bick^2^, Edward Purssell^3^, Lindsay Mallick^4^, Cath Taylor^5^

#### ^1^Florence Nightingale Faculty of Nursing, Midwifery & Palliative Care, Kings College London, London, UK; ^2^ Department of Women and Children’s Health, School of Life Course Sciences, Faculty of Life Sciences and Medicine, King’s College London, UK ; ^3^ School of Health Sciences, City, University of London, London, UK; ^4^ Avenir Health, Rockville, MD, USA ; ^5^ School of Health Sciences, University of Surrey, UK

**Correspondence:** Kimberly Peven (Kimberly.peven@kcl.ac.uk)

**Background**

In Malawi, most newborns have contact with health service in the first days of life, however, coverage of comprehensive newborn care remains low. National efforts have been made to improve and scale up provider training in newborn care. We aimed to evaluate the associations between provider training in newborn care and interventions implemented in practice at facilities in Malawi.

**Methods**

Using data from the 2013-14 Service Provision Assessment survey in Malawi, we employed ordinal and logistic regression models to evaluate the associations between training and observed newborn interventions, adjusting for facility and provider factors. Data included 1294 providers, 527 facilities and 474 observed births.

**Results**

Newborn care providers reported attending trainings covering on average 4.7 (95%CI=4.5,5.0) themes in their career and 2.6 (95%CI=2.4,2.8) themes in the past two years out of 14 training themes included in the survey (Figure 1). Following births observed at facilities, providers implemented on 6.9 (95%CI=6.6,7.2) of 12 recommended interventions. Preliminary analyses did not show an association between number of recent training themes attended by a provider and the number of interventions implemented after birth (AOR=1.05,95%CI=0.98,1.12), however for every additional recent training theme attended, providers were 8% (AOR=1.08,95%CI=1.02,1.16) more likely to implement more than the mean number of interventions.

**Conclusions**

While newborn care providers in Malawi reported attending recent trainings on newborn care, the relationship between recent training and improved practice is not clear. More research is required to understand drivers of improved newborn care following provider training and scale-up coverage of comprehensive newborn care.

**Figure 1 (abstract 23).**
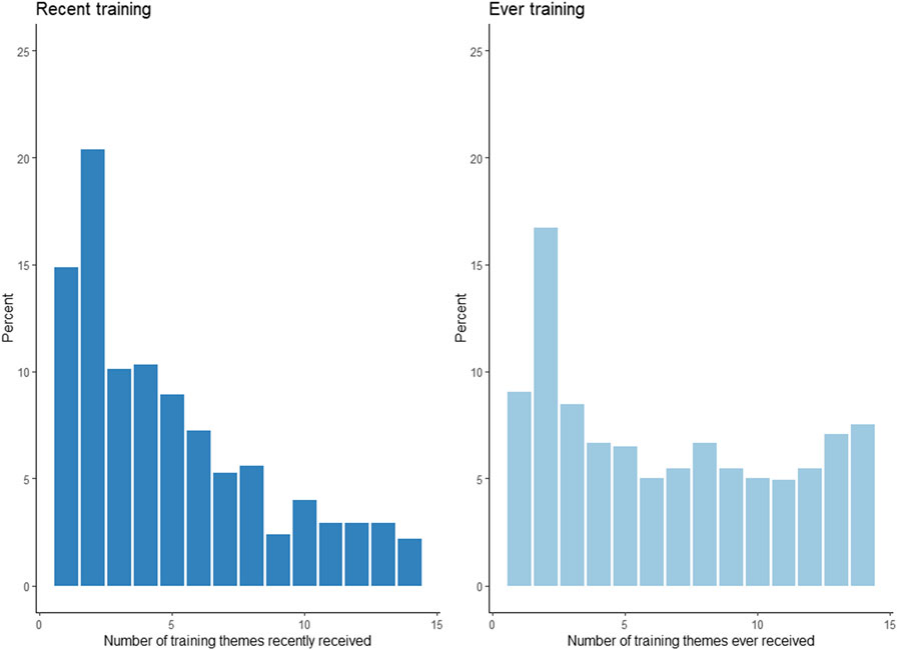
Number of newborn care training themes received among providers ever having received newborn care training

## O24 Knowledge brokering in a public mental health program: does sharing knowledge derived from implementation science help to improve outcomes?

### Kristian G. Hudson^,^ Rebecca Lawton^,^ Siobhan Hugh-Jones

#### School of Psychology, University of Leeds, Leeds, UK

**Correspondence:** Kristian G. Hudson (kgharvey@gmail.com)

**Background**

Schools need mental health interventions that are deliverable, scalable and effective. However, rates of successful implementation of evidence-based practices in schools is low. An underpinning factor is lack of understanding, and attention, by commissioners, providers and schools, to evidence about what works to bolster effective and sustained implementation. Knowledge brokering is one way to improve use of implementation science in program delivery in complex settings. To date, evidence for the use of KBs in public health settings is scarce, and their use for brokering *implementation* related knowledge is non-existent.

The aim of this study was to examine whether it was feasible and acceptable for knowledge about implementation to be brokered to a steering group responsible for a wide scale, public mental health, school-based intervention, and what impact this might have on their implementation decisions.

**Methods**

The primary researcher attended 13 (3 hrs each) monthly steering group meetings during the initial implementation phase of the public mental health program and shared implementation knowledge with them.

With consent, meeting notes were taken by the primary researcher, and a journal kept of 12 meetings; 5 meetings were also audio recorded and SG minutes referred to. All data were amalgamated and ordered into month-by-month summaries. The analysis attempted to identify when implementation knowledge was shared with the group (coded a ‘key moment’) and what the steering group did with this knowledge (coded a ‘key outcome’).

**Results**

Over the 13 meetings, 15 key moments led to 14 key outcomes, 10 of which involved implementation decisions being made based on brokered knowledge about implementation science (Figure 1). Some brokered knowledge was not acted upon.

**Conclusions**

Knowledge brokering on implementation science to a group responsible for public mental health is feasible and acceptable, although this may have been promoted by the strong research culture amongst steering group members.

**Figure 1 (abstract 24).**
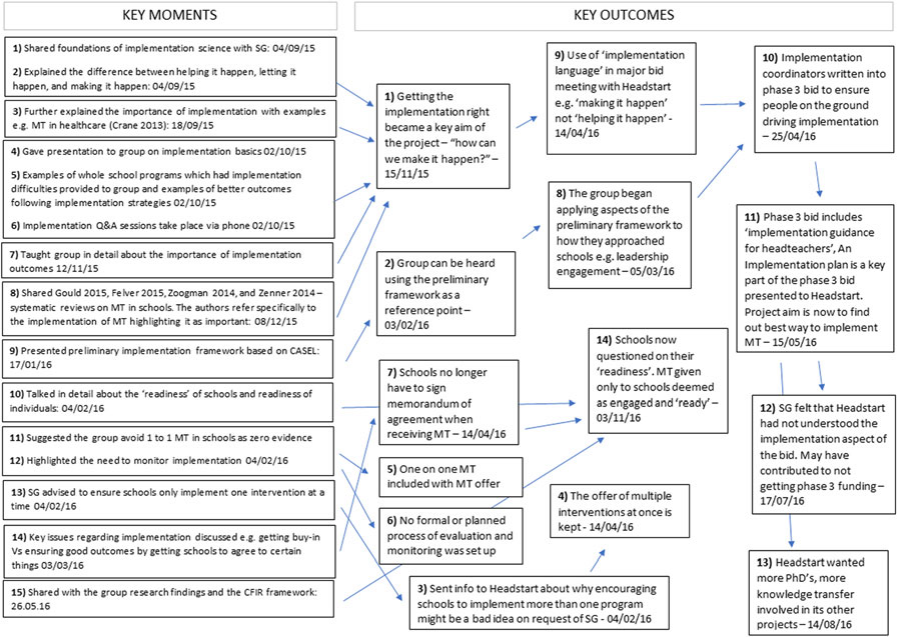
Key moments and Key Outcomes from sharing implementation science knowledge with the SG

## O25 Withdrawn

## P26 Withdrawn

## P27 Rapid testing of service innovations in general practice: an evaluation of the Primary Care Home model in Newham

### Darren Sharpe and Lauren Herlitz

#### Institute for Health and Human Development, University of East London, Water Lane, London, UK

**Correspondence:** Lauren Herlitz (l.herlitz@uel.ac.uk)

**Background**

In Autumn 2018, Newham Clinical Commissioning (CCG) group piloted two service enhancements to groups of six general practices under the national Primary Care Home (PCH) programme: 1) managing in-hours demand for doctors’ appointments through online triage, care navigation, and additional GP hours shared between practices, and 2) a complex case (CC) management team for patients with multiple co-morbidities. The evaluation aimed to assess the feasibility and acceptability of the PCH model and identify factors that could affect the roll-out of services across the Borough.

**Method**

A mixed-methods, formative process evaluation was carried out between December 2018 and May 2019: 1) routinely collected data, 2) interviews and focus groups with practitioners, deliverers and a patient participation group, 3) anonymised patient questionnaires, 4) web analytics, 5) document analysis. Normalization Process Theory [1] informed the development of research tools and analysis.

**Results**

In relation to managing in-hours demand, practices utilised to capacity the additional GP hours provided by locum doctors; this service enhancement was well-received by practices and patients alike. The implementation of online triage and care navigation was mixed, working most successfully in practices that were fully committed to embedding the approach and tackling challenges that arose. Although the CC management service was viewed positively by attending patients, particularly in addressing unmet social care needs, there was low uptake of the service by patients and carers, and practices did not ‘buy-in’ to the service within the pilot timeframe.

**Conclusion**

Within six months, the tested PCH models helped to widen access to GPs and stimulated partnership working ahead of the implementation of mandated Primary Care Networks, as envisaged in the NHS Long Term Plan. Securing the commitment of practices to joint working and ongoing communication work between all stakeholder groups was crucial to the success of the model.

**Acknowledgements**

This evaluation was funded by NHS Newham Clinical Commissioning Group and was supported by the National Institute for Health Research (NIHR) Collaboration for Leadership in Applied Health Research and Care North Thames at Bart’s Health NHS Trust (NIHR CLAHRC North Thames). The views expressed in this article are those of the author(s) and not necessarily those of the NHS, the NIHR, or the Department of Health and Social Care

**Reference**

1. May C, Finch T. Implementing, embedding, and integrating practices: an outline of Normalization Process Theory. Sociology. 2009; 43(3):535-554.

## P28 Developing and scaling up a strategy for patient and public involvement in the Centre for Implementation Science and King’s Improvement Science

### Len Demetriou^*1*^, Josephine Ocloo^*2*^, Lucy Goulding^*1*^, Barbora Krausova^*1*^ & Louise Hull^*3*^

#### ^1^King’s Improvement Science, Institute of Psychiatry, Psychology and Neuroscience, King’s College London, Denmark Hill, London, UK; ^2^Centre for Implementation Science, King’s College London, Denmark Hill, London, UK; ^3^Centre for Implementation Science & King’s Improvement Science, King’s College London, Denmark Hill, London, UK

**Correspondence:** Len Demetriou (helena.demetriou@kcl.ac.uk)

**Background**

The Centre for Implementation Science (CIS) and King’s Improvement Science (KIS) carried out patient and public involvement (PPI) activities separately until 2017 when both teams identified the need for more consistent models of PPI, so merged efforts to develop an overarching PPI strategy for the Centre that would be co-produced with members of the public living and/or using health services in South London.

The first workshop in September 2017 brought together 17 members of the public and 17 Centre staff to answer the question **‘How can we best include and involve people in the work of the CIS and KIS?’**

This poster describes how we have scaled-up our PPI strategy, what we have achieved, and lessons learnt.

**Method**

Ideas from the initial workshop were developed and scaled up using several methods to co-produce our PPI strategy.

These include:

Consensus building methodology (used to facilitate the first workshop)

Focus groups and consultation meetings to action and develop ideas collaboratively

Ensuring we strive to include diverse members of the public throughout all stages

**Results**

Our achievements showcase how we have implemented our PPI strategy from ‘ideas’ to actionable achievable results.

These include:

Setting up the Public Involvement Advisory Panel to offer expertise and advise from public members to staff on projects within the Centre

Two public members recruited to our working group which oversees the operational running of the strategy

A monthly Involvement Bulletin to keep in contact with public members, build positive working relationships and offer opportunities to get involved

A guidance document being co-developed to offer advice and information for staff in the Centre on how best to carry out PPI activities

**Conclusion**

We have developed multiple ways to include public voices throughout the work of the Centre, striving for parity between academic and public members and we continue to move forward with public involvement that is not tokenistic, but trying to reach ‘beyond the usual suspects’ [1].

**Acknowledgements**

We would like to thank everyone, past and present, who have been involved in developing the strategy. We are especially grateful to all our public members for their time, invaluable expertise and contributions and for sharing their experiences and knowledge with us.

**Reference**

1. Beresford, P. 2013, Beyond the Usual Suspects. Shaping Our Lives Press.

## P29 Theoretical Domains Framework (TDF) and COM-B model based implementation study of a school-based, toothbrushing complex intervention

### Min-Ching Wang^1,2^, Wei Han Chen ^2^

#### ^1^Department of Stomatology, Taipei Veterans General Hospital, Taipei, Taiwan; ^2^ Department of Dentistry, National Yang-Ming University, Taipei, Taiwan

**Correspondence:** Min-Ching Wang (showmerry@hotmail.com)

**Background**

The school-based supervised toothbrushing program with fluoride toothpaste twice a day medical demand in the most deprived cohort of children. Health inequalities were decreased as well [1]. However, the implementation of a school-based brushing program is not merely based on oral hygiene instructions. Knowledge transfer has shown little effect on behaviour change. Changing behaviour requires an understanding of the context which behaviours occur [2]. The multifactorial nature of human behaviour made it difficult to evaluate this program through traditional randomized controlled trials. Therefore, we used a qualitative study design to analyse the potential facilitators and barriers in this complex intervention program [3].

**Method**

Focus groups and semi-structure interviews were used to collect the ideas from the elementary school students, teachers, and staff in this program. The topic guides were developed according to the Theoretical domains framework combined with COM-B Model. Data were analyzed with a content analysis approach.

**Results**

A sustainable environment which could encourage brushing behaviour was important. Toothbrush and toothpaste need to be supplied, and a set brushing schedule should be arranged. Brushing became a daily routine. Students may supervise each other brushing without the teachers’ help. Opportunity in COM-B model, instead of motivation or capability, was the key theme to implement the school-based brushing program.

**Conclusion**

Future studies will re-evaluate the program and develop a logic model in order to transfer the toothbrushing program to other schools.

**Acknowledgements**

This publication is independent research. The school-based toothbrushing program is funded and supported by Taiwan Academy of Pediatric Dentistry, and the Give2Asia organization

**References**

1. Anopa Y, McMahon AD, Conway DI, Ball GE, McIntosh E, Macpherson LM. Improving Child Oral Health: Cost Analysis of a National Nursery Toothbrushing Programme. PloS one. 2015;10(8):e0136211.

2. Michie S, Johnston M, Abraham C, Lawton R, Parker D, Walker A. Making psychological theory useful for implementing evidence based practice: a consensus approach. Quality and Safety in Health Care. 2005;14(1):26-33.

3. Atkins L, Francis J, Islam R, O'Connor D, Patey A, Ivers N, et al. A guide to using the Theoretical Domains Framework of behaviour change to investigate implementation problems. Implement Sci. 2017;12(1):77.

## O30 Use of the World Health Organisation Surgical Safety Checklist in Low and Middle Income Countries: A Systematic Review of Implementation Strategies Used and Outcomes Reported

### Olivia Clancy^1^, Ijeoma Okonkwo^1^, Kimberly Peven^2^, Nick Sevdalis^2^, Andy Leather^3^, Stephanie Russ^2^ and Michelle White^1,2, 3^

#### ^1^Department of Anaesthesia, Great Ormond Street Hospital, London, UK; ^2^Centre for Implementation Science, Kings College London, London, UK; ^3^Centre for Global Health and Health Partnerships, Kings College London, London, UK

**Correspondence:** Olivia Clancy (livclancy7@gmail.com)

**Background**

The WHO Surgical Safety Checklist (SSC) reduces surgical morbidity and mortality by 50% but is not widely used in low and middle income countries (LMICs). Evidence of the effectiveness of SSC implementation in LMICs is lacking. Therefore, we aimed to systematically identify all SSC implementation studies in LMICs, the implementation strategies used, and critically evaluate success based on implementation outcomes.

**Method**

We used the PICO framework to design a systematic review of SSC implementation strategies and reported outcomes in LMICs, registering the study on PROSPERO. 73 strategies and 8 outcomes were pre-defined according to the Expert Recommendations for Implementing Change and Proctor’s implementation outcome framework respectively.

**Results**

We identified 1562 articles and included 45 in analysis. In preliminary analysis: mean number of strategies employed was 6 (range=0-24); most commonly from the “train and educate stakeholders” cluster (*n=29)* and least commonly from the “engage consumers” cluster (*n=0) (Figure 1).* Most commonly reported implementation outcomes were fidelity and penetration. Average penetration was 85% (95%CI=77%,90%) and average fidelity (adherence to basic safety measures) was 82%(95%CI=76%, 86%). SSC use was associated with reduced mortality (RR=0.72,95%CI=0.63,0.82) and morbidity (RR=0.58,95%CI=0.47,0.72). Mean implementation strategy importance ratings were associated with increased fidelity and penetration but this was not statistically significant.

**Conclusion**

This systematic review identified wide variation in implementation strategies used and outcomes reported in LMICs. Successful implementation showed a non-statistically significant association with mean strategy importance rating, as well as an association with improved clinical outcomes.

**Figure 1 (abstract 030).**
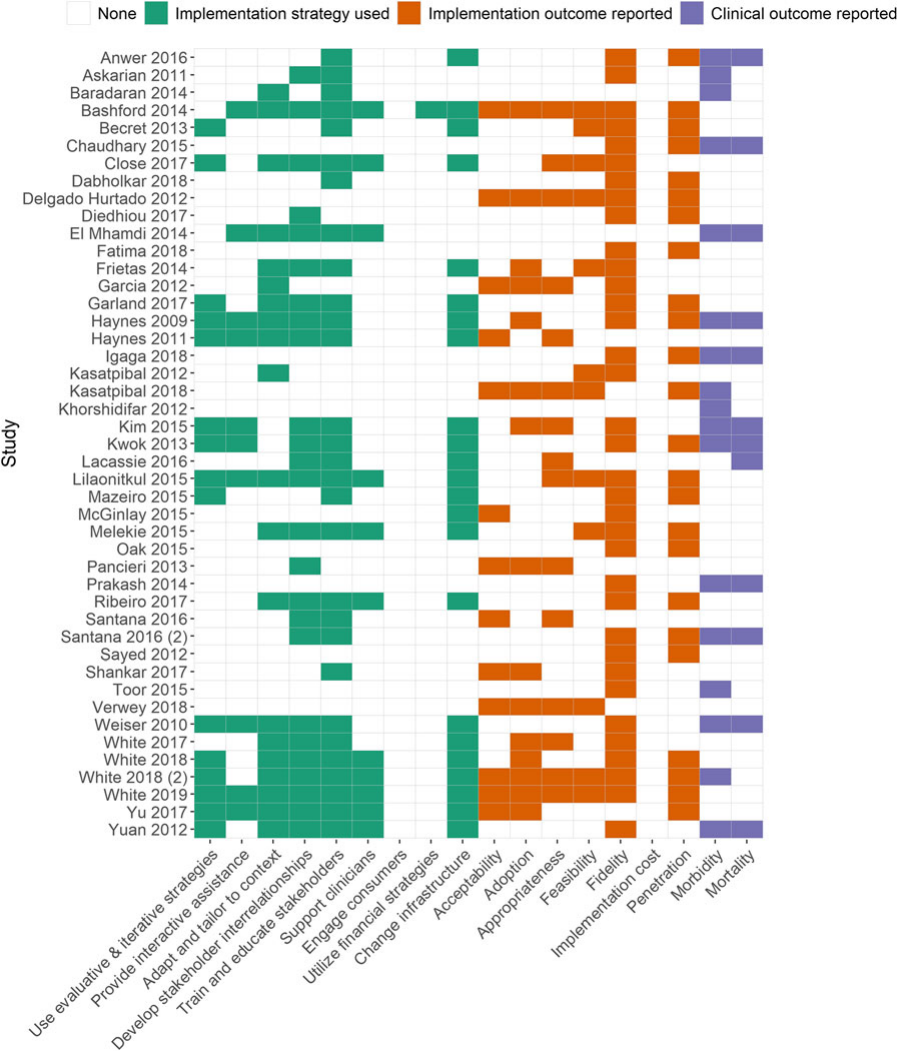
Implementation strategies, implementation outcomes, and clinical outcomes

## O31 Scaling up the Obstetric Anal Sphincter Injury Care Bundle – Evaluation of clinical and implementation effectiveness of a national quality improvement project to reduce severe perineal trauma sustained during childbirth

### Posy Bidwell^1^, Ranee Thakar^2^, Ipek Gurol-Urganci^3^, Louise Silverton^4^, Alexandra Hellyer^1^, Vivienne Novis^1^ and Nick Sevdalis^5^

#### ^1^ Clinical Quality, Royal College of Obstetricians and Gynaecologists, London, UK; ^2^ Obstetrics and Gynaecology, Croydon University Hospital, London, UK; ^3^ Department of Health Services Research and Policy, London School of Hygiene and Tropical Medicine, London, UK; ^4^ Royal College of Midwives, London, UK; ^5^ Health Service & Population Research, King’s College London, London, UK

**Correspondence:** Posy Bidwell (pbidwell@rcog.org.uk)

**Background**

Obstetric anal sphincter injuries (OASI) can severely affect quality of life. In England OASI rates amongst primiparous women tripled from 1.8% in 2000 to 5.9% in 2011 [1]. Aetiology is multifactorial, however, contributing factors are training inconsistencies, lack of awareness and variation in practice [1-3]. To address this, the OASI Care Bundle was developed and supported by the Royal College of Obstetricians and Gynaecologists (RCOG) and the Royal College of Midwives (RCM).

**Method**

Using a stepped-wedged trial design the intervention was sequentially rolled out every three months to four regions across England, Scotland and Wales [4]. Each region comprised of four maternity units of various sizes and types. Local clinical champions (midwives and obstetricians) cascaded training and educational materials within their units. Implementation was evaluated using mixed methods. A Theory of Change was developed to map how the desired reduction in OASI rates was expected to occur. Patient-level data (October 2016–April 2018) was collected to determine OASI rates pre- and post- intervention. A qualitative process evaluation examined acceptability, feasibility and sustainability for clinicians and women.

**Results**

The OASI rate was 3.3% pre- and 3.0% post-intervention (adjusted OR 0.79 (0.65-0.97), p=0.03). Most barriers to adoption of the intervention had corresponding enablers. Data capabilities varied, affecting ability to measure compliance. A heavy training burden was placed on champions, who had little or no dedicated time for this. Challenging autonomy and perceptions of women’s wishes created resistance amongst some clinicians. Success drove further uptake.

**Conclusion**

Change takes time. Complex topics require good communication and good feedback mechanisms. Appetite for change and senior buy-in facilitate engagement. Service-user support is a powerful enabler and women’s involvement is key. Our project increased team cohesion, which was strengthened by top-level support from the two Royal Colleges. This project offers a blueprint for effective scale-up of improvement interventions within maternity.

**Acknowledgements**

Local champions were key to success of the OASI Care Bundle and we are grateful to them for all their hard work and dedication to improving outcomes for women. The OASI Care Bundle was fully funded by the Health Foundation.

**Trial Registration**

The OASI Care Bundle is registered on the ISRCTN registry 10.1186/ISRCTN12143325

**References**

1. Gurol-Urganci, I., et al., Third- and fourth-degree perineal tears among primiparous women in England between 2000 and 2012: time trends and risk factors. BJOG, 2013. 120(12): 1516-25.

2. Laine, K., et al., Incidence of obstetric anal sphincter injuries after training to protect the perineum: cohort study. BMJ Open, 2012. 2(5).

3. Naidu, M., A. Sultan, and R. Thakar, Reducing obstetric anal sphincter injuries using perineal support a preliminary experience. Int Urogynecol J, 2017. 28: 381-389

4. Bidwell, P. et al. A multi-centre quality improvement project to reduce the incidence of obstetric anal sphincter injury (OASI): study protocol. BMC Pregnancy Childbirth. 2018. 18(1):331

## O32 Evaluating the uptake of evidence-based guidance and associated determinants to inform implementation strategies that reduce maternal and perinatal morbidity in pregnant women with chronic hypertension: a mixed-method multi-centre study

### Rebecca Whybrow^1^, Joanna Girling^2^, Heather Brown^3^, Hannah Wilson^1^, Louise Webster^1^, Jane Sandall^1^, Lucy Chappell^1^

#### ^1.^ Department of Women and Children’s Health, King’s College London, London, UK; ^2.^ Chelsea and Westminster NHS Trust, London, UK; ^3.^ Brighton and Sussex University NHS Trust, East Sussex, UK.

**Correspondence:** Rebecca Whybrow (Rebecca.whybrow@kcl.ac.uk)

**Background**

Mortality from hypertensives disorders is reducing [1] but morbidity is prevalent [2]. Uptake of evidence-based guidelines and the associated determinants is unknown.

**Method**

Convergent parallel multi-method evaluation of evidence uptake and integrated analysis to inform implementation strategy. National survey, case-notes review, observations and qualitative interviews at 3 NHS Trusts (2018).

Results

Survey (n=97)

Self-reported compliance with guidelines was high. Absence of anti-hypertensive prescribing guidance was mimicked by variance in reported practice.

Case-notes (n=55)

100% cessation of teratogenic medication thereafter variance in first-line anti-hypertensive prescribing existed. Target setting documentation occurred infrequently (56.4%) however action was taken when blood-pressure exceeded 150/100mmHg (76.5%) resulting in internalised setting (20%) and absent setting (23.5%). 45.5% of women developed severe hypertension.

Observations (n=23)

Provision of correct amount and type of information to support shared decision-making (52%). Women’s involvement in decisions (43%) and offered choice of anti-hypertensive (19%).

Women’s interviews (n=18)

14/18 experienced conflict about hypertension management, conflict resulted in 8/18 non-adhering (fig 1.a). Facilitators to concordant adherence were found to be determined by ‘trust’ mediated by management of side effects, information and professional’s approach to women (fig 1.b).

Clinician’s interviews (n=13)

Sub-optimal information was attributed to strength of anti-hypertensive evidence and lack of guidance. Clinicians also lacked SDM skills and tools. Optimal pathways and schedules of care are unknown.

**Conclusion**

Severe hypertension is prevalent. Strategies to reduce associated morbidity should engender ‘trust’ and improve target setting. Standardised pathways and schedules of care are required, accompanied by research into safety and effectiveness of anti-hypertensive.

**Acknowledgements**

National Institute for Health Research (NIHR) Research professorship (RP- 2014-05-019)

NIHR CLAHRC (South London) Maternity and Women’s Health.

A special thank you to all the women who took part in the study giving their time and sharing their experiences.

**Trial Registration**

South London Ethics Committee 17/LO/2041

**References**

1. Knight M, Nair M, Tuffnell D, Kenyon S, Shakespeare J, Brocklehurst P, Kurinczuk JJ (Eds.) on behalf of MBRRACE-UK. Saving Lives, Improving Mothers’ Care − Surveillance of maternal deaths in the UK 2012−14 and lessons learned to inform maternity care from the UK and Ireland Confidential Enquiries into Maternal Deaths and Morbidity 2009−14. Oxford: National Perinatal Epidemiology Unit, University of Oxford; 2016 [www.npeu.ox.ac.uk/mbrrace-uk].

2. Bramham K, Parnell B, Nelson-Piercy C, Seed PT, Poston L, Chappell LC. Chronic hypertension and pregnancy outcomes: systematic review and meta-analysis. BMJ : British Medical Journal. 2014;348:g2301.

**Figure 1 (abstract O32).**
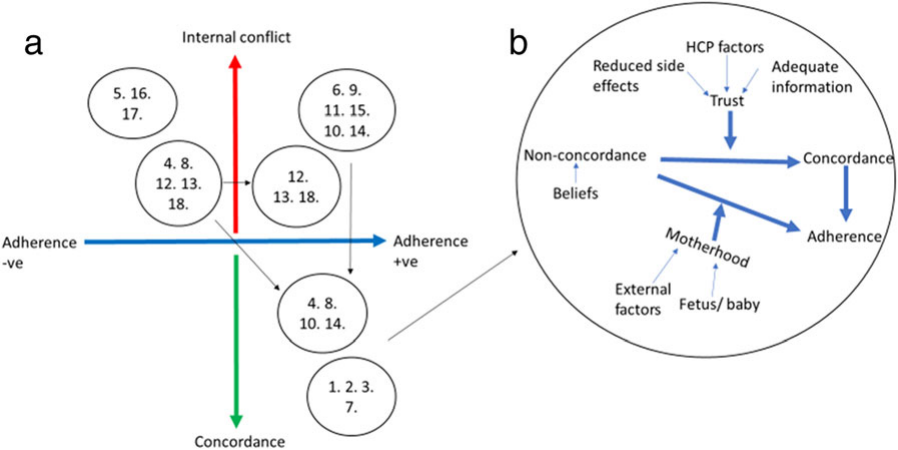
a. Adherence and concordance with prescribed AHTs in women with chronic hypertension. 1b. Facilitators of adherence and of concordance

## O33 Scale-up of SmartVA, an electronic decision support tool to improve cause of death certification in The Philippines

### Rohina Joshi^1,2^, Lene Mikkelsen^3^, Ferchito Avileno^4^, Agnes Segarra^4^, Alan D Lopez^3^

#### ^1^The George Institute for Global Health, Sydney, Australia; ^2^School of Public Health and Community medicine, University of New South Wales, Sydney, Australia; ^3^University of Melbourne, Melbourne, Australia; ^4^Department of Health, Manila, Philippines.

**Correspondence:** Rohina Joshi (rjoshi@georgeinstitute.org)

**Background**

The majority of deaths in Philippines occur out-of-hospital and require a medical certificate of cause of death (MCCD) by Municipal Health Officers (MHOs). In order to improve the quality of MCCD of home deaths, SmartVA auto-analyse, an electronic decision support tool for physicians was introduced to assist MHOs in the certification process.

**Method**

SmartVA Auto-analyse was implemented in a phased manner. A stakeholder consultation was followed by site visits and workshops with MHOs. A pre-test was conducted to assess feasibility and acceptability by MHOs in 13 municipalities. The intervention included training MHOs in SmartVA Auto-analyse, and MCCD (Fig 1). Next, the intervention was scaled-up to 50 municipalities across 6 regions. A mixed-methods evaluation was performed using cause of death data and group discussions with the MHOs and policy meeting with the Department of Health.

**Results**

During the pilot 5649 home deaths were registered (54% male). SmartVA used to certify 81% of all deaths (4333), for the remaining 19%, doctors could assign a cause of death based on the availability of medical records. Physicians agreed with the SmartVA diagnosis in 65% cases. Group discussions identified two key themes 1) SmartVA was acceptable to MHOs, and 2) It standardised certification for home deaths and improved the quality of death certification. The Government plans to roll out the intervention in a phased manner over the next 2 years.

**Conclusion**

SmartVA can be implemented by MHOs and improve the quality of death certification of home deaths in Philippines.

**Acknowledgements**

The project was funded by Bloomberg Philanthropies and the Department of Foreign Affairs, Australia. We acknowledge the Country Coordinator, the Staff from the Department of Health, the implementing partners, all the participating Municipal Health Officers, and the respondents.

**Figure 1 (abstract O33).**
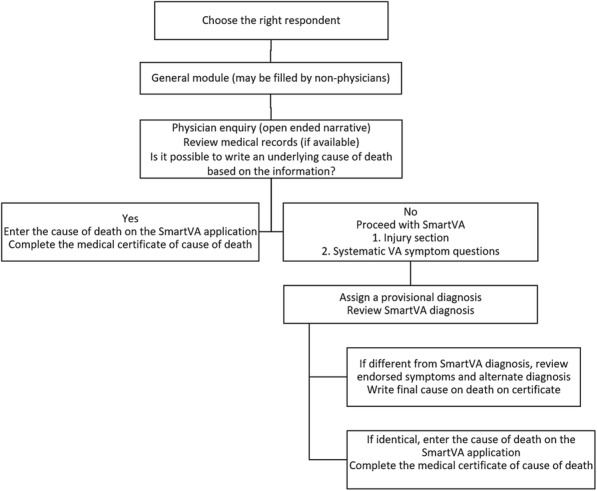
Standard operating procedure for use of SmartVA Auto-analyse by physicians

## O34 A systematic review of the implementation outcome instruments used in healthcare settings and their measurement properties

### Sabah Boufkhed^1^, Zarnie Khadjesari^1,2^, Silia Vitoratou^3^, Laura Schatte^1^, Alexandra Ziemann^1,5^, Christina Daskalopoulou^4^, Eleonora Uglik-Marucha^3^, Nick Sevdalis^1^, Louise Hull^1^

#### ^1^ Centre for Implementation Science, Health Service and Population Research Department, King’s College London, UK;^2^ School of Health Sciences, University of East Anglia, UK; ^3^ Biostatistics and Health Informatics Department, King’s College London, UK; ^4^ Health Service and Population Research Department, King’s College London, UK; ^5^ Centre for Healthcare Innovation Research, City, University of London, UK

**Correspondence:** Sabah Boufkhed (sabah.boufkhed@kcl.ac.uk)

**Background**

The use of validated instruments is needed to measure the implementation of evidence-based interventions and services [^1,2^]. To help researchers and practitioners identify relevant tools to measure implementation outcomes in physical healthcare settings, we conducted a systematic review aiming to identify quantitative implementation outcome measures, and critically appraise their psychometric properties [^3^].

**Method**

We searched seven databases (MEDLINE, EMBASE, PsycINFO, HMIC, CINAHL and the Cochrane Library), from inception to March 2017. A search update (March 2017-18) is currently ongoing. Eligible studies assessed measurement properties of implementation outcome instruments in physical healthcare settings. Proctor et al. ’s framework [^4]^ was used to select the implementation outcomes: *acceptability, appropriateness, feasibility, adoption, penetration, implementation cost and sustainability*. We used the COnsensus-based Standards for the selection of health Measurement INstruments (COSMIN) checklist to assess their methodological quality [^5^] and a newly developed Contemporary Psychometrics (ConPsy) checklist to assess their robustness.

**Results**

Two reviewers independently screened 6,586 titles and abstracts, and 313 full papers. More than 50 publications were included, among which over half measured acceptability. Less than 10 studies measured each of the other implementation outcomes (figures pending update). Most studies reported some measurement properties. Content and structural validity were the most reported properties for the validity domain; and internal consistency for the reliability domain. Studies had overall poor or fair methodological quality, and the ConPsy checklist indicated poor instruments’ robustness. However, some acceptability measures, like the Ottawa acceptability of decision rules instrument [^6^ ], or the Impact of Health Information Technology Scale [^7^ ], had excellent methodological quality.

**Conclusion**

This review provides a much-needed repository of appraised implementation outcome instruments for physical healthcare settings. It highlights the efforts to quantify implementations outcomes, and the need for further psychometric testing of existing measures. It also encourages researchers to consider how to best measure feasibility, penetration, implementation cost and sustainability.

**Acknowledgments**

The research is supported by the National Institute for Health Research (NIHR) Collaboration for Leadership in Applied Health Research and Care South London (CLAHRC South London) at King’s College Hospital NHS Foundation Trust.

Sabah Boufkhed and Zarnie Khadjesari received funding from the King’s Improvement Science (KIS), which is a part of the NIHR CLAHRC South London. KIS is funded by King’s Health Partners, Guy’s and St Thomas’ Charity, the Maudsley Charity and the Health Foundation.

**References**

1. Lewis CC, Fischer S, Weiner BJ, Stanick C, Kim M, Martinez RG. Outcomes for implementation science: an enhanced systematic review of instruments using evidence-based rating criteria. Implement Sci. 2015;10:155. doi:10.1186/s13012-015-0342-x

2. Clinton-McHarg T, Yoong SL, Tzelepis F, et al. Psychometric properties of implementation measures for public health and community settings and mapping of constructs against the Consolidated Framework for Implementation Research: a systematic review. Implementation Science. 2016;11:148. doi:10.1186/s13012-016-0512-5

3. Khadjesari Z, Vitoratou S, Sevdalis N, Hull L. Implementation outcome assessment instruments used in physical healthcare settings and their measurement properties: a systematic review protocol. BMJ Open. 2017;7(10):e017972. doi:10.1136/bmjopen-2017-017972

4. Proctor E, Silmere H, Raghavan R, et al. Outcomes for implementation research: conceptual distinctions, measurement challenges, and research agenda. Adm Policy Ment Health. 2010;38(2):65–76. doi:10.1007/s10488-010-0319-7

5. Terwee CB, Mokkink LB, Knol DL, Ostelo RW, Bouter LM, de Vet HC. Rating the methodological quality in systematic reviews of studies on measurement properties: a scoring system for the COSMIN checklist. Qual Life Res. 2011;21(4):651–657. doi:10.1007/s11136-011-9960-1

6. Brehaut JC, Graham ID, Wood TJ, Taljaard M, Eagles D, Lott A, Clement C, Kelly AM, Mason S, Kellerman A, Stiell IG. Measuring acceptability of clinical decision rules: validation of the Ottawa acceptability of decision rules instrument (OADRI) in four countries. Medical Decision Making. 2010 May;30(3):398-408.

7. Dykes PC, Hurley A, Cashen M, Bakken S, Duffy ME. Development and psychometric evaluation of the Impact of Health Information Technology (I-HIT) scale. Journal of the American Medical Informatics Association. 2007 Jul 1;14(4):507-14.

## P35 The role of Principal Investigators of research studies in the adoption and scaling up of healthcare interventions

### Sal Stapley, Rob Anderson

#### University of Exeter College of Medicine and Health, Devon, UK

**Correspondence:** Sal Stapley (s.a.stapley@exeter.ac.uk)

**Background**

The importance of assessing the impact of universities’ research is gaining importance with an increase in impact weighting in case studies from the 2014 to 2021 REF exercises. Similarly funders require Principal Investigators (PIs) to describe impact pathways and statements for grant applications to demonstrate how research may be expected to inform policy or practice, and ultimately provide benefit to society. Historically the primary role of the academic has been to carry out rigorous and original research to add to disciplinary knowledge. This study aims to examine the role of the PI to describe the role of PIs in driving the adoption and uptake of their own research findings in the healthcare setting.

**Method**

Purposive sampling of the 163 REF 2014 impact case studies in Unit of Assessment 2 (Public Health, Health Services and Primary Care) was used to identify case studies, which cited a definitive effectiveness trial in the impact summary. Trial PIs will be emailed a questionnaire to describe the roles and work in driving the uptake of their research findings (or intervention). They will also be asked about what perceived freedoms/enablers or disincentives/barriers meant they have been able to do more/less impact generation than they would have liked, the role of serendipity/luck, how their role in enabling implementation has changed over time, and whether they made use of specific tools or frameworks to guide their evidence implementation efforts.

**Results**

Data will be analysed and presented using descriptive statistics.

**Conclusion**

The study will characterise the variety of roles that lead researchers can play in promoting the successful uptake or application of their research findings in health policy and practice, together with outlining the perceived contextual factors that have enabled (or constrained) them to adopt such roles.

## P36 Withdrawn

## P37 Implementation of an online healthy weight toolkit to support practitioners working with pre-school children

### Hassan, S.M^1.2^, Nugent, M^3^, Bradbury, D^4.5^, Watson, P.M^4^.

#### ^1^NIHR Collaboration for Leadership in Applied Health Research and Care North West Coast (CLAHRC NWC); ^2^The University of Liverpool, Liverpool, UK; ^3^Early Help, Public health, Blackburn with Darwen, Lancashire, England; ^4^Physical Activity Exchange, School of Sport and Exercise Sciences, Liverpool John Moore’s University, UK; ^5^School of Psychology, University of Worcester, UK.

**Correspondence:** Hassan, S. M (s.m.hassan@liverpool.ac.ukc)

**Background**

Recent data shows Blackburn with Darwen (BwD) has a higher than average prevalence of both over- and under-weight in children starting school. Following local concern for the lack of pre-school child weight management provision, an 8-module online health weight toolkit was co-produced in partnership between local stakeholders and academic partners. All modules include guidance from the Royal College of Paediatrics and Child Health (RCPCH), National Institute for Health and Care Excellence (NICE), World Health Organisation (WHO), and NHS. As part of the Partner Priority Programme (PPP) of the Collaboration for Leadership in Applied Health Research and Care North West Coast (CLAHRC-NWC), an implementation strategy was developed that focuses on engaging healthcare professionals with the online healthy weight intervention tool.

**Method**

The implementation strategy draws on the Consolidated Framework for Implementation Research (CFIR) and involves multidisciplinary collaboration between academics, healthcare professionals, local authority commissioners and four public members. This presentation will reflect on the CFIR process and milestones in the implementation of the online tool. The shared learning will offer insight into how stakeholder engagement enhances the process of implementation.

**Results**

The process in engaging different stakeholders in the development of an implementation strategy consolidates the acknowledgment of local expertise. However, this process can be challenging when considering the inner and outer context in creating a strong partnership for implementation. For example it required ensuring shared vision, addressing team dynamics, alignment with local pathways, exploring individual role restrictions, and alignment with national policy/guidance.

**Conclusion**

The CFIR provided a structured framework that enabled exploration of different factors to support the process of engaging different stakeholders and public members in the development of an implementation strategy.

**Acknowledgments**

We would like to acknowledge the tremendous work of Dr Ruth Young in supporting the implementation of this project. We would also like to show gratitude to Shirley Goodhew for supporting the internship process of this project.

## O38 Embedding implementation science in practice: learning from the Partner Priority programme

### Shaima Hassan^1,2^, Cheryl Simmill-Binning*^1,3^, Alison McCracken^4^, Tamsin Cripps^4^, Annette O’Donoghue^4^, Jane Tomlinson-Wright^4^, Clarissa Giebel^1,2^

#### ^1^NIHR Collaboration for Leadership in Applied Health Research and Care North West Coast (CLAHRC NWC); ^2^The University of Liverpool, Liverpool, UK; ^3^Lancaster University, Bailrigg, Lancaster, UK; ^4^University Hospital of Moorecambe Bay NHS Foundation Trust, Cumbria, UK

**Correspondence:** Shaima Hassan (s.m.hassan@liverpool.ac.uk)

**Background**

Increasingly, practitioners and other professionals are expected to be involved in research and evaluations not only as advisers but also as researchers. The Collaboration for Leadership in Applied Health Research and Care North West Coast (CLAHRC NWC) Partner Priority Programme (PPP) has undertaken an approach of work that facilitated this type of co-working, which in its third iteration set out to build capacity in Implementation Science. This offered a Consolidated Framework for Implementation Research (CFIR) through which practitioners could consider the drivers associated with the implementation of their projects.

**Method**

Four reflective narratives explore the journey travelled by practitioners new to the framework, reflecting the inside and outside setting, characteristics of the intervention and the individuals involved. Offering guidance as to how in the future their fellow professional could be engaged and enabled to deliver Implementation Science in their institutions.

**Results**

Being at the forefront of change is not easy, however, the CFIR offered the practitioners a means to discuss and understand the challenges they were facing in the implementation of their projects. No longer was it seen as a case of individually named people appearing to be obstructive. It became apparent that changes were challenging their knowledge and beliefs about the intervention as the new project did not sit easily with their understanding of the wider organisation. The characteristics of one project was found to be a major challenge not only in terms of delivery but also as a concept and practice within mainstream health care.

**Conclusion**

Practitioners need support to enable them to apply Implementation Science frameworks such as the CFIR. In particular, they need a learning environment in which to apply the methodology, reflect on the outcome, and assess the impact whilst continuing with the ‘day job’.

## O39 Experience of using mental health indicators in six low and middle-income countries where mental health is integrated in primary care: a qualitative study

### Shalini Ahuja^1^, Charlotte Hanlon^2,1^, Dan Chisholm^3^, Maya Semrau^4,1^, Dristy Gurung^5^, Jibril Abdulmalik^6^, James Mugisha^7^, Ntokozo Mntambo^8^, Fred Kigozi^7^, Inge Petersen^8^, Rahul Shidhaye^9^, Nawaraj Upadhaya^5^, Crick Lund^10,1^, Sara Evans-Lacko^11,1^, Graham Thornicroft^1^, Oye Gureje^6^, Mark Jordans^1,5^

#### ^1^Centre for Global Mental Health, Institute of Psychiatry, Psychology and Neuroscience, King’s College London, UK; ^2^Charlotte Hanlon, Addis Ababa University, Addis Ababa, Ethiopia; ^3^Department of Mental Health and Substance Abuse, World Health Organization, Switzerland; ^4^ Global Health and Infection Department, Brighton & Sussex Medical School, Brighton, UK; ^5^Transcultural Psychosocial Organization, Nepal; ^6^Department of Psychiatry, University of Ibadan; ^7^Butabika National Referral and Teaching Mental Hospital, Uganda; ^8^University of Kwazulu-Natal, South Africa; ^9^Public Health Foundation of India; ^10^Alan J Flisher Centre for Public Mental Health, Department of Psychiatry and Mental Health, University of Cape Town, South Africa; ^11^Personal Social Services Research Unit, London School of Economics and Political Science

**Correspondence:** Shalini Ahuja (shalini.ahuja@kcl.ac.uk)

**Background**

Efforts to scale up integrated mental health care in low and middle-income country are underway. Some of these initiatives are focusing on strengthening information systems for mental health. In the previous publications, we described the need and the process of implementing key indictors covering mental health service delivery in lower and middle-income countries. These measures were introduced by a multi country consortium Emerald (Emerging Mental Health System) at a district level in six LMICs (Ethiopa, India, Nepal, Nigeria, South Africa and Uganda).

**Methods**

In this paper, through a qualitative study with 128 in depth interviews, we assessed the feasibility, acceptability and utility of a set of indicators for routine monitoring of mental health care and evaluated the performance of a set of indicators for routine monitoring of mental health care.

**Results**

Most mental health indicators were deemed relevant and potentially useful for improving care, and therefore acceptable to end users. Exceptions were indicators on functionality, cost and severity. The simplicity of the data capturing formats contributed to the feasibility of using forms to generate data on mental health indicators. Health workers reported increasing confidence in their capacity to record the mental health data and minimal additional cost to initiate mental health reporting. However, overstretched primary care staff and the time-consuming reporting process affected perceived sustainability.

**Conclusion**

This qualitative study provides a potential model for evaluating the use of mental health information systems across low and middle income countries and recommends key feasible indicators to be adapted for similar settings.

## O40 Amalgamating theoretical frameworks to understand individual and organisational level implementation

### Stephanie Best^1,2^, Janet C Long^1^, Melissa Martyn^3,4^, Clara Gaff^3,4^, Jeffrey Braithwaite^1^, Natalie Taylor^5^

#### ^1^Australian Institute of Health Innovation, Macquarie University, Sydney, Australia; ^2^Australian Genomic Health Alliance, Murdoch Children’s Research Institute, Melbourne, Australia; ^3^Dept. of Paediatrics, University of Melbourne, Melbourne, Australia; ^4^Melbourne Genomics Health Alliance, Walter and Eliza Hall Institute, Melbourne, Australia; ^5^New South Wales Cancer Council, Woolloomooloo, Sydney, Australia.

**Correspondence:** Stephanie Best (stephanie.best@mq.edu.au)

**Background**

Despite the rapid advances in genomic medicine, few studies incorporate implementation science theoretical frameworks.^1^ When exploring implementation on multiple levels, for example the use of clinical genomics in Australia, it can be challenging to identify a single appropriate approach and amalgamating frameworks may be advantageous. We used two frameworks to learn from early adopters to design interventions to support initial implementation and sustainability of genomics in wider and less genomically specialised healthcare settings.

**Method**

Working with Australian Genomics and Melbourne Genomics, we interviewed 37 people with experience of clinical genomic testing. i) non-genetic medical specialists, using the Theoretical Domains Framework (TDF) to understand determinants of practice along the patient journey ii) service-level decision makers, using the Translation Science to Impact framework (TSci) to understand organisational and external factors affecting translation phases. Individual and organisational barriers and enablers were coded to the TDF and the TSci, respectively. Areas for overlapping themes across frameworks were explored.

**Results**

Clinician level barriers and enablers were identified across four key target areas (patient identification, test selection, communicating results, and mainstreaming) along the genomics pathway and represented all of the TDF domains. Service-level factors were identified to represent all the TSci Key Issues across the pre-adoption, adoption, implementation and sustainability phases relating to ‘Needed Practice Supports’. Overlap between frameworks was found across all target areas, but predominantly for clinician-identified barriers and enablers to mainstreaming of genomics which covered all TSci phases.

**Conclusion**

Our study highlights the potential to combine different theoretical frameworks to maximise the value of theoretical approaches in highly complex settings. The next steps will be to explore the extent to which a theory driven approach to intervention design (e.g., behaviour change techniques) can be applied to address both individual and organisational level barriers.

**Reference**

^1^ Roberts et al The current state of implementation science in genomic medicine: opportunities for improvement. Genet Med [Internet]. 2017;19(8):858–63. Available from: http://www.nature.com/doifinder/10.1038/gim.2016.210

## P41 Implementing rapid genomic testing in acute paediatric care in Australia

### Stephanie Best^1,2^, Janet C Long^1^, Jeffrey Braithwaite^1^, Natalie Taylor^3^, Belinda Maclaren^2,4^, Helen Brown^5^, Sebastian Lunke^6^, Zornitza Stark^2,6^

#### ^1^Australian Institute of Health Innovation, Macquarie University, Sydney, Australia; ^2^Australian Genomic Health Alliance, Murdoch Children’s Research Institute, Melbourne, Australia; ^3^New South Wales Cancer Council, Woolloomooloo, Sydney, Australia; ^4^Dept. of Paediatrics, University of Melbourne, Melbourne, Australia; ^5^Melbourne Genomics Health Alliance, Walter and Eliza Hall Institute, Melbourne, Australia; ^6^Victoria Clinical Genetics Service, Murdoch Children’s Research Institute, Melbourne, Australia.

**Correspondence:** Stephanie Best (stephanie.best@mq.edu.au)

**Background**

Genomic testing, reading the entire genetic information, provides an opportunity to end the diagnostic journey for many patients with rare disease. Typically, return of results takes around six months – a timeline too slow for the management of acutely-unwell children. Whilst faster turnaround times can deliver more clinically useful results, it demands a restructure of the whole genomics pathway and causes disruption to traditional practices.

Melbourne Genomics led a rapid-genomic testing (<21days) real-world project in two hospitals. We used implementation science principles to learn from this project and apply them in an Australian Genomics multi-centre project for ultra-rapid genomic testing (<5days) in acutely-unwell children.

**Method**

Data on the barriers and enablers to implementation were gathered (observations at multidisciplinary team meetings, research journals (February 2016-September 2017) from the project leads and participating genetic clinical and laboratory staff. The multi-centre project data collection also included interviews with laboratory scientists, genetic clinicians and paediatric intensivists (April-June 2019). Analysis was undertaken using the Consolidated Framework for Implementation Research (CFIR).

**Results**

Several CFIR constructs were prominent (see table 1) across both hospitals. Enablers were identified, and outcomes noted.

**Conclusion**

Accounting for organisational culture and tailoring implementation science principles to real-world problems are key to success. Using implementation science theory has enabled structured learning from one project and facilitated the initiation of a multi-centre project that will inform the next steps of delivering genomic testing in the acute paediatric setting

**Reference**

1. Stark et al, Meeting the challenges of implementing rapid genomic testing in acute pediatric care Genetics in Medicine, 2018; *20*(12), 1554-1563.

**Table 1 (abstract P41).** Example CFIR construct, barriers, solutions, and outcome (adapted from Stark et al^1^)

CFIR construct	Barrier	Enabler identified	Outcome
Networks & Communications	Delay in referral to clinical genetics	Clearly communicate availability of rapid genomic testing and indications for referral	Earlier referrals to clinical genetics
Relative advantage		Ensure referrers get feedback on diagnostic and clinical impact of rapid genomic testing, e.g. at monthly NICU/Genetics case review meetings	Increased number of referrals to clinical genetics
Implementation climate	Parental difficulty processing complex information in a stressful environment	Increased genetic counselling support in the acute setting	Higher rate of rapid genomic testing acceptance by families
Adaptability	Results batched ready for weekly dedicated MDT meeting	Initiate multidisciplinary team meetings for single cases, with minimum quorum defined	Reduction in time to report
Formal implementation leaders	Absence of an established rapid genomic testing pathway	Clinical and laboratory champions identified	Formal rapid genomic testing operating procedure established

Interviews for the multi-state trial are ongoing. Early findings indicate the importance of relationships between genetic and paediatric intensivist staff

## P42 Social prescribing for people with motor neurone disease in Liverpool: Enabling access to local well-being activities to prevent social isolation and mental ill health

### Suzanne Simpson^1^, Sandra Smith^2^, Moira Furlong^2,3^, Janet Ireland^4^, Clarissa Giebel ^2,5^

#### ^1^ The Walton Centre NHS Trust; ^2^ NIHR CLAHRC NWC; ^3^ The Motor Neurone Disease Association; ^4^ The Brain Charity; ^5^ Institute of Population Health Sciences, University of Liverpool

**Correspondence:** Suzanne Simpson (suzanne.simpson@thewaltoncentre.nhs.uk)

**Background**

Motor neurone disease (MND) affects around 5,000 people in the UK. This project aims to identify potential services and activities provided by the third sector in the Sefton and Liverpool area that can support psychological wellbeing in people living with MND (plwMND).

**Method**

This project is part of the NIHR CLAHRC NWC Partner Priority Programme, enabling partner organisations to implement services collaboratively with academics and the public. The project team includes three public advisors, two with experience of caring for someone with MND, and one working for a third sector organisation providing information and advice services for individuals with neurological conditions. Recruitment is carried out by MND association visitors, outpatient and community therapists. To ensure the occupation is based on the person’s interests, the Modified Interest Checklist is used [1]. Once suitable activities are identified, the project lead attends the activities with the plwMND, acting as a support for the plwMND and the activity provider for a total of six weeks.

Referrers complete questionnaires prior to recruitment to establish their current use of wellbeing services, and quality of life measures are administered to the plwMND and their family carer pre and post intervention. Interviews will be carried out post intervention with plwMND and their family carer, the referrers, and community providers.

**Results**

Starting in February, two plwMND have utilised the service so far. Both have recently finished working and expressed concern that they had no structure or purpose. They have chosen varied activities including reading and history groups, art classes, bird watching, and accessible exercise.

**Conclusion**

Currently, we are at the beginning of this implementation project. Findings will have important implications on how a social prescribing service can be provided to plwMND, and if successful, how this service can be rolled out wider across the region and nationally.

**Acknowledgements** This study was *part-funded by The National Institute for Health Research Collaboration for Leadership in Applied Health Research and Care North West Coast (NIHR CLAHRC NWC). The views expressed here are those of the author(s) and not necessarily those of the NHS, the NIHR, or the Department of Health and Social Care.*

**Reference**

1. Taylor, RR, Kielhofner, G. *Kielhofner’s model of human occupation: theory and application*. 5^th^ ed. Philadelphia: Wolters Kluwer; 2017.

## P43 Stakeholder-driven, mixed-methods implementation evaluation of two psychoeducational programmes to reduce the incidence of severe hypoglycaemia in type 1 diabetes

### Tayana Soukup^1^, Louise Hull^1^, Ioannis Bakolis^1,2^, Andy Healey^1^, Nick Sevdalis^1^, Lived experience group^2^

#### ^1^Centre for Implementation Science, King’s College London, UK; ^2^Department of Biostatistics and Health Informatics, King’s College London, UK; ^2^People With Diabetes Group: Arthur Durrant, Mike Kendall, Victoria Ruszala, Mel Stephenson, Lis Warren, London UK

**Correspondence:** Tayana Soukup (tayana.soukup@kcl.ac.uk)

**Background**

In line with the Medical Research Council’s (MRC) recommendations for evaluating complex interventions [1-3], we have set out to evaluate implementation of two novel psychoeducational courses for people with type 1 diabetes (T1D) and problematic hypoglycaemia. The courses are delivered within a hybrid type 2 effectiveness-implementation cluster randomised trial (4; NCT02940873). Here we report co-design of the implementation arm of the trial.

**Method**

This was an international, multisite study conducted October-December 2017 across 5 diabetes centres in England, UK, and Massachusetts, US.

We conducted 11 focus groups with overall 28 intervention stakeholders, including, individuals with lived experience of T1D and hypoglycaemia unawareness (n=6 attending 2x1.5hrs focus groups), and healthcare professionals (HCPs; n=22; physicians, nurses, dietitians, psychologists and support staff attending 9x1hr focus groups).

We were guided by the tool for designing implementation research [5-6], including also:

- MRC framework [1-3],
- Reach Effectiveness Adoption Implementation Maintenance [7],
- Consolidated Framework for Implementation Research [8-9],
- Implementation outcomes by Proctor et al [10], and
- Implementation strategies compendium by Powel et al [11].

In a qualitative manner, the stakeholders reviewed for relevance, feasibility and clarity a selection of outcomes, tools [12], approaches, measurement time-points and participant groups. Following each meeting, stakeholders’ feedback was incorporated in an iterative manner – until the final versions of the materials were produced for implementation within the trial.

**Results**

Stakeholder input has enabled relevant, feasible, and appropriate implementation outcomes, validated surveys, interview questions, participant groups, and measurement time-points to be identified. The final outcomes included acceptability, feasibility, appropriateness, fidelity, context, unintended consequences and implementation cost. Mixed-methods approach was deemed most acceptable.

**Conclusion**

Our study offers a stakeholder-driven methodological approach to co-designing an evaluation of complex intervention implementation within a hybrid trial. Input from HCPs and people with lived experience, who co-authored this work, continues to be instrumental in maximising relevance of this research.

**Acknowledgements**

We would like to thank the representatives of the people with type 1 diabetes, as well as the health care professionals involved, for their time and commitment in the design of this study protocol.

**References**

1. Medical Research Council. Developing and evaluating complex interventions: new guidance. 2006; https://www.mrc.ac.uk/documents/pdf/complex-interventions-guidance/.

2. Moore GF, Audrey S, Barker M, Bond L, Bonnel C., Hardeman W, Moor L, O’Cathain A, Tinati T., Wight D, Bair J. Process evaluation of complex interventions: Medical Council guidance. BMJ. 2015; doi:10.1136/bmj.h1258

3. Craig P, Dieppe P, Macintyre S, Michie S, Nazareth I, Petticrew M. Developing and evaluating complex interventions: the new medical research council guidance. Int J Nurs Stud. 2013;50:587-592;p.591.

4. Amiel S, Choudhary P, Jacob P, Smith E, de Zoysa N, Gonder-Frederick L, Kendall M. et al. A group randomised controlled trial of a novel intervention addressing cognitions in adults with treatment-resistant problematic hypoglycaemia complicating type 1 diabetes therapy – the Hypoglycaemia Awareness Restoration Programme for people with type 1 diabetes and problematic hypoglycaemia persisting despite optimised self-care (HARPdoc). BMJ Open. [in press]

5. Implementation Science Research Development (ImpRes) tool and guide. http://www.kingsimprovementscience.org/files/ImpRes_Guide_May2018_2.pdf. Accessed 03 August 2018.

6. Hull L, Goulding L, Khadjesari Z, Davis R, Healey A, Bakolis I, Sevdalis N. Designing High-Quality Implementation Research: Development, Application and preliminary evaluation of the Implementation Science Research Development (ImpRes) tool and guide. Implement Sci. [in press]

7. Harden SM, Smith ML, Ory MG, Smith-Ray R, Estabrooks PA, Glasgow RE. Re-aim in clinical, community, and corporate settings: perspectives, strategies, and recommendations to enhance public health impact. Front Public Health. 2018; doi.org/10.3389/fpubh.2018.00071

8. Kirk AM, Kelley C, Yankey N, Birken SA, Abadie B, Damschroder L. A systematic review of the use of the Consolidated Framework for Implementation Research. Implementation Sci. 2016;11:72.

9. Damschroder LJ, Hagedorn HJ. A guiding framework and approach for implementation research in substance use disorders treatment. Psychol Addict Behav. 2011;25(2):194.

10. Proctor E, Silmere H, Raghavan R, Hoymand P, Aarons G, Bunger A, Griffey R, Hensley M. Outcomes for implementation research: conceptual distinctions, measurement challenges, and research agenda. Adm Policy Ment Health. 2011;38(2):65-76.

11. Powell BJ, Waltz TJ, Chinman MJ, Damschroder LJ, Smith JL, Matthieu MM, Proctor EK, Kirchner JE. A refined compilation of implementation strategies: results from the Expert Recommendations for Implementing Change (ERIC) project. Implementation Sci. 2015;10:21.

12. Weiner BJ, Lewis CC, Stanick C, Powell BJ, Dorsey CN, Clary AS, Boynton MH, Halko H. Psychometric assessment of three newly developed implementation outcome measures. Implement Sci. 2017;12:108.

**Table 1 (abstract O43).** Stakeholder-driven data collection plan for the implementation study: assessment objectives, data, instruments, timeline and participants

#	Study outcomes	Definition of the study outcome	Data type	Data collection method	Measurement time-point	Stakeholder groups*
IMPLEMENTATION OUTCOMES:						
1.	Acceptability [8]	Extent to which programme is perceived to be agreeable and acceptable for hypoglycaemia and diabetes management.	Quantitative	AIM† survey	Post-intervention	HCPs, people with T1D and their relatives
			Qualitative	Interview	Post-intervention	
2.	Appropriateness [8]	Extent to which programme is perceived to be fit and relevant for hypoglycaemia and diabetes management.	Quantitative	IAM‡ survey	Post-intervention	HCPs, people with T1D and their relatives
			Qualitative	Interview	Post-intervention	
3.	Feasibility [8]	Extent to which programme can be successfully used or carried out to reduce incidents of severe hypoglycaemia.	Quantitative	FIM§ survey	Post-intervention	HCPs, people with T1D and their relatives
			Qualitative	Interview	Post-intervention	
4.	Fidelity of delivery [8]	Extent to which programme is delivered as intended.	Quantitative	Checklist	Post-intervention	Diabetes educators and psychologists
5.	Fidelity of receipt [8]	Extent to which programme is received as intended.	Qualitative	Interview	Post-intervention	People with T1D
6.	Adoption [8]	Intention to adopt and use the knowledge and skills learned in the programme in everyday hypoglycaemia and diabetes management.	Qualitative	Interview	Post-intervention	HCPs, people with T1D and their relatives
7.	Sustainability [8,12]	Facilitators and barriers to sustained use of the programme.	Qualitative	Interview	Post-intervention	HCPs, people with T1D and their relatives
8.	Implementation costs [8,12]	Costs associated with prospective implementation of the programme.	Qualitative	Interview	Post-intervention	HCPs, people with T1D and their relatives
OTHER OUTCOMES:						
9.	Unintended consequences of programmes [9]	Positive or negative consequences that are not anticipated at the time of programme implementation.	Qualitative	Interview	Post-intervention	HCPs, people with T1D and their relatives
10.	Contextual factors [9]	Facilitators and barriers to the implementation of the programme.	Qualitative	Interview	Post-intervention	HCPs, people with T1D and their relatives
11.	Implementation strategies [11]	Strategies used to deliver and implement the programme; they refer to methods or techniques to enhance and promote adoption, implementation and sustainability of the programme.	Qualitative	Interview	Post-intervention	HCPs

*Note*. *HCPs = Health Care Professionals incl. diabetes educator, physician, psychologist, and administrative support. †AIM = Acceptability of Intervention Measure [12]; ‡IAM = Intervention Appropriateness Measure [12]; §FIM = Feasibility of Intervention Measure [12]

## O44 Looking into determinants and mechanisms related to the implementation of a pain management guideline in nursing homes

### Thekla Brunkert^1^, Michael Simon^1,2^, Franziska Zúñiga ^1^

#### ^1^ University of Basel, Department Public Health (DPH), Nursing Science (INS), 4056 Basel, Switzerland; ^2^ Inselspital Bern University Hospital, Nursing Research Unit, 3010 Bern, Switzerland.

**Correspondence:** Thekla Brunkert (Thekla.brunkert@unibas.ch)

**Background**

Underutilization of evidence-based pain management in nursing homes is common. Evidence towards effective approaches to improve adoption of evidence-based practices in nursing homes is limited. Furthermore, little is known about the mechanisms of how implementation strategies affect practice change [1]. This study uses a mixed-methods approach to explore the mechanisms and determinants related to the implementation of a pain management guideline in Swiss nursing homes.

**Method**

We conducted a process evaluation alongside an effectiveness-implementation study in four nursing homes. Based on an a priori contextual analysis in the participating homes, we developed a conceptual framework describing the hypothesized interactions of implementation strategy, contextual factors, mechanisms, and implementation outcomes. With a care worker questionnaire survey at baseline (n=136), after three (n= 99) and six months (n=83) we assessed our hypothesized central mechanism, self-efficacy in pain management, and self-reported guideline adoption. We computed linear mixed-effect models to assess changes over time in self-efficacy and logistic regressions to assess associations between self-efficacy and guideline adoption. Concurrently, we conducted focus groups with care workers (n=16) to gain a deeper understanding of the implementation process, interactions between potential mechanisms, determinants and outcomes of the implementation. Qualitative data was analysed using a deductive thematic analysis approach.

**Results**

Overall, there was a significant increase in self-efficacy after three and six months (p<0.001). Self-reported adoption of guideline components ranged between 44% and 73%. We found significant associations between self-efficacy and adoption of some guideline components, e.g., performing a comprehensive pain assessment.

Qualitative findings indicate a different response to implementation strategies by registered nurses and nursing aides, e.g., in role empowerment. Training workshops increased awareness towards pain, stimulating changes in pain assessment behaviour.

**Conclusion**

Understanding implementation processes in a multilevel context is challenging. Developing a conceptual framework can facilitate the exploration of potential mechanisms and influences of contextual factors.

**Reference**

1. Lewis CC, Klasnja P, Powell BJ, Lyon AR, Tuzzio L, Jones S, et al. From Classification to Causality: Advancing Understanding of Mechanisms of Change in Implementation Science. Frontiers in Public Health. 2018; 6:136

## P45 Impact of evidence-based healthcare education for Chinese medicine practitioners: A pre-post evaluation

### Charlene HL Wong^1,2^, Irene XY Wu^3^, William KW Cheung^4^, Robin ST Ho^4^, Matthew J Leach^5,7^, Wenbo Peng^5^, Yan Zhang^5,8^, Justin CY Wu^1,2^, Vincent CH Chung^4,6^

#### ^1^Department of Medicine and Therapeutics, The Chinese University of Hong Kong, Hong Kong; ^2^Hong Kong Institute of Integrative Medicine, The Chinese University of Hong Kong, Hong Kong; ^3^Xiangya School of Public Health, Central South University, Changsha Hunan, China; ^4^Jockey Club School of Public Health and Primary Care, The Chinese University of Hong Kong, Hong Kong; ^5^Australian Research Centre in Complementary and Integrative Medicine (ARCCIM), Faculty of Health, University of Technology Sydney, Sydney, Australia; ^6^School of Chinese Medicine, The Chinese University of Hong Kong, Hong Kong; ^7^Department of Rural Health, Division of Health Sciences, University of South Australia, Adelaide, Australia; ^8^Division of Integrative Medicine, School of Medicine, Texas Tech University Health Sciences Center, Texas, USA.

**Correspondence:** Vincent CH Chung (vchung@cuhk.edu.hk)

**Background**

WHO Traditional Medicine Strategy 2014-23 recommended evidence-based healthcare (EBHC) education for traditional and complementary medicine (T&CM) professionals, including Chinese medicine practitioners (CMPs). We evaluated the impact of a customized educational workshop on Hong Kong CMPs’ knowledge, attitude and practice of EBHC.

**Method**

Two validated instruments, Evidence-based Practice Questionnaire (EPQ) and Evidence-based Practice Inventory (EPI), were used to assess the impact of EBHC education. Paired t-tests were used to compare scores before and after the workshop. Multiple linear regression was performed to explore the associations between changes in EPQ/EPI scores and CMPs’ characteristics.

**Results**

CMPs who completed the workshop (n=59) demonstrated significant improvements in the attitude (p=0.013) and knowledge domains of the EPQ (p=0.005). Significant improvements were also observed in the attitude, perceived behavioural control, decision making, and intention and behaviour domains of the EPI. CMPs who had never received prior EBHC training showed a larger magnitude of improvement in the EPI attitude (p=0.032), decision making (p=0.015), and intention and behaviour (p=0.015) domains post-workshop.

**Conclusion**

Our findings suggest that tailored workshop is effective in strengthening knowledge and in improving attitudes towards EBHC. Future initiatives consider incorporating this education approach into CMP curricula, as well as facilitating implementation of EBHC in routine Chinese medicine practice**.**

## O46 Chinese medicine practitioners’ beliefs and attitudes in applying evidence-based synopses in routine practice – a qualitative study

### Charlene HL Wong^1,2^, Vincent CH Chung^3,4^, Justin CY Wu^1,2^, Per Nilsen^5^

#### ^1^Department of Medicine and Therapeutics, The Chinese University of Hong Kong, Hong Kong; ^2^Hong Kong Institute of Integrative Medicine, The Chinese University of Hong Kong, Hong Kong; ^3^Jockey Club School of Public Health and Primary Care, The Chinese University of Hong Kong, Hong Kong; ^4^School of Chinese Medicine, The Chinese University of Hong Kong, Hong Kong;^5^Division of Community Medicine, Department of Medical and Health Sciences, Linköping University, Linköping, Sweden.

**Correspondence:** Vincent CH Chung (vchung@cuhk.edu.hk)

**Background**

The WHO Traditional Medicine Strategy 2014-23 has been promoting the development of complementary medicine using evidence-based healthcare (EBHC) approach. The Hong Kong government has adopted this approach to ensure safety, quality and efficacy of Chinese medicines. However, little is known about the perspectives of Hong Kong Chinese medicine practitioners (CMPs) on EBHC. We aimed to examine key facilitators and barriers of applying results from evidence-based synopses in routine practice among Hong Kong CMPs.

**Method**

Four synopses with the summary of systematic reviews and randomized controlled trials on Chinese medicine interventions were presented to CMPs (n=15). Qualitative face-to-face interviews on their views on these synopses were then conducted. A semi-structured interview guide was designed based on the Consolidated Framework for Implementation Research (CFIR). CFIR was also used as a framework to guide the analysis by means of categorizing barriers and facilitators of applying results from evidence-based synopses in CMPs’ routine practice.

**Results**

Analysis of the interview identified seventeen constructs in four CFIR domains: intervention characteristics, outer setting, inner setting and characteristics of individuals. Facilitators which promoted the application of evidence-based synopses in

CMPs’ routine practice included adaptability, cost, patient needs and resources, external policy and incentives. Barriers of the intervention included complexity and available resources. Constructs that acted as both facilitators and barriers included intervention source, evidence strength and quality, relative advantage, peer pressure, structural characteristics, compatibility, access to knowledge and information, knowledge and beliefs about the intervention, self-efficacy, individual identification with organization and culture.

**Conclusion**

This study distinguished a number of CFIR constructs and illustrated how they influenced the application of results from evidence-based synopses in routine practice among Hong Kong CMPs. Findings have indicated the importance of providing continuing education on EBHC for CMPs which may facilitate the integration of clinical research evidence into their routine practice.

## P47 Bridging communication gap in HIV treatment programs through Closed user group (CUG) mobile technology: Approach to HIV patient monitoring and referrals services on the Strengthening integrated delivery of HIV/AIDS services project

### Yemisi Ogundare^1^, Olamide Agbaje^1^, Idongesit Utuk^1^, Chidubem Momah^1^, Chinedu Agbakwuru^1^ Ezekiel James^2^, Oluyinka Olayemi^1^, Hadiza Khamofu^1^ Satish Raj Pandey^1^

#### ^1^ FHI 360, Abuja, Nigeria; ^2^ USAID, Abuja, Nigeria

**Correspondence:** Yemisi Ogundare (yogundare@aahnigeria.org)

**Background**

Timely and complete referrals for HIV clients in need of care within health facilities, facility-community and inter facility is crucial in closing the gaps for HIV care. Monitoring, tracking and routine interaction with HIV clients by health workers and case managers has been proven to reduce leakages in treatment cascades and improve retention in care. However, most health facilities in Nigeria are challenged by the non-existence of communication infrastructure or resources required to facilitate timely tracking and completion of referrals for HIV positive clients. The USAID-funded Strengthening integrated delivery of HIV/AIDS services (SIDHAS) project implemented the provision of closed user group (CUG) mobile technology as a strategy to closing the gap.

**Description**

SIDHAS procured 2,606 mobile phones and lines, distributed to 785 health facilities, laboratories and 28 community-based organizations across 13 states in Nigeria. Mobile networks were distributed according to the strength of network available in the locale. The CUG allowed health workers providing HIV care and treatment services to call each other within the network with zero charges and to follow up HIV clients referred within service delivery points and those referred to other health facilities to ensure complete referrals. A desk review was conducted on the CUG directory and communication logs for 4 states between June – December 2017.

**Lessons learned**

Review of the phone directory and call logs shows evidence of inter and intra facility communications, therefore contributing to improved referral and linkage rate from 39% to 74% within the period for some states. Data collected from 4 states also showed about 2240 defaulters returned within the period compared to 785 that returned to care before the CUG implementation. Overall, Improvement in completion rates for referrals and tracking and provision of adherence support to HIV clients was recorded.

**Conclusions/Next steps**

Given the results demonstrated by removing communication barriers and bridging the gap through CUG innovation, expanding CUG distribution to support groups in communities can be explored to reducing stigma and closing communication gaps amongst infected individuals.

## O48 Review of Strengthening Integrated delivery of HIV/AIDs project (SIDHAS) Accountability framework of HIV/AIDS financing with civil society organizations, government agencies and private institutions in Nigeria

### Yemisi Ogundare^1^, Olamide Agbaje^1^, Idongesit Utuk^1^, Chidubem Momah^1^, Nuhu Aliyu^1^, Clement Okhakemen^1^, Charles Esuga^1^, Oluyinka Olayemi^1^, Hadiza Khamofu^1^, Ezekiel James^2^, Satish Raj Pendey^1^

#### ^1^ FHI 360, Abuja, Nigeria; ^2^ USAID, Abuja, Nigeria

**Correspondence:** Yemisi Ogundare (yogundare@aahnigeria.org)

**Background**

HIV care and treatment in Nigeria is largely managed by a combination of public and private health facilities. Although these institutions have experienced declines in funding from government/private owners over the years, they still account for the majority of patients in care. To bridge the funding gap, the USAID-funded SIDHAS project introduced different types of grants financing approaches and accountability frameworks to support scale up of HIV prevention, care and treatment services in 13 states in Nigeria. We share experiences from the various accountability frameworks applied on the project.

**Description**

From inception of SIDHAS project in 2011, FHI360 Nigeria conducted thorough pre-award assessments, after which 76 sub-awards were executed in 13 states between SIDHAS and State government for public health, faith-based, civil society organization, and private health facilities. Capacity building through trainings and mentoring was conducted. Accounting practices such as system enhancement, compliance audits, quarterly grant modification, expenditure analysis, scorecard audit, fund request review, documentation and reporting was mandated for each implementing agency (IA) routinely. A qualitative content analysis of the IAs’ accounting practices, frameworks, reports and HIV prevention, care and treatment services delivered was conducted between October 2016 and September 2017.

**Lessons Learnt**

From the monthly expenditure analysis, sub-award monitoring reports and HIV program data reported across the 76 IAs showed that 100% of the IAs were compliant with submission of financial and program reports for the period of assessment. HIV testing, initiation of client on treatment and provision of care services were not interrupted at those sites within the period. An analysis of this result further shows that the combination of capacity building and use of accounting frameworks was instrumental to the results. About 82% Improvement in quality of program and financial reports received was recorded within the year demonstrating improved technical, institutional and financial domains of sustainability.

**Conclusion**

A combination of accountability approaches resulted in improved ability of IAs to provide HIV services, manage, and account for donor resources. Providing a combination of accountability structures for IAs can expand their ability to better manage and sustain delivery of comprehensive HIV prevention, care and support services.

## P49 Lessons learnt from a novel HIV care & treatment funding model for health facilities in Nigeria, a resource constrained country: The reimbursement for incidentals (RFI) funding model

### Yemisi Ogundare^1^, Olamide Agbaje^1^, Irene Osaigbovo^1^, Idongesit Utuk^1^, Chidubem Momah^1^, Chinedu Agbakwuru^1^, Oluwasanmi Adedokun^1^ Ezekiel James^2^, Oluyinka Olayemi^1^, Hadiza Khamofu^1^

#### ^1^ FHI 360, Abuja, Nigeria; ^2^ USAID, Abuja, Nigeria

**Correspondence:** Yemisi Ogundare (yogundare@aahnigeria.org)

**Background**

Partnership between the governments of Nigeria (GoN) and the United States (USG) through PEPFAR aims to strengthen the capacity to scale up HIV prevention, care and treatment services in Nigeria. While PEPFAR streamlined operational health facility costs in 2014, the GoN was unable to adequately fund operational HIV/AIDS related services. As a result, health workers were using their personal funds to pay for operational costs, and when they could not, services were not administered. In response to these gaps in care, the SIDHAS project developed the RFI funding model to better cover health facility operational costs. The RFI model reimburses health facilities through service providers, for operational cost incurred during service delivery. This abstract describes lessons learnt using the RFI funding model.

**Description**

The RFI funding model for 13 SIDHAS-supported states in Nigeria was developed to cover operational health facility costs such as telephone cards and home visits for HIV clients, internet costs for data entry, transport for sample logging to hub laboratories, and quality improvement measures. Funds reimbursed for operation costs to each health facility were calculated at 110 naira ($0.31 US) per client currently on ART in comprehensive treatment facilities and 300 naira ($0.83 US) per pregnant woman tested for HIV in PMTCT standalone facilities. The model reimbursed health workers monthly using standardized verifiable activity request and report forms.

**Lessons learned**

The RFI funding model was adopted in 100% of health facilities across 13 SIDHAS supported states in Nigeria. Health workers were motivated because of the RFI model directly reimburses cost incurred during service delivery. Improvement in service delivery was recorded, resulting in more clients visiting health facilities. Monthly pay out to health facilities improved from an average of 57% in 2015, to 66% in 2016 and 84% in 2017.

**Conclusions**

The RFI model has proven to be a motivational tool for health workers and a means to fund and improve HIV care treatment related operational costs in a resource-constrained country such as Nigeria.

